# Supplementary material for: Adapting, piloting, and evaluating a pediatric lead screening and risk-reduction intervention in Nairobi: A hybrid implementation-effectiveness trial protocol
Source: PLoS One. 2026 Jun 4;21(6):e0349153. doi: 10.1371/journal.pone.0349153 (PMC13235933; doi:10.1371/journal.pone.0349153)
Supplement: S2 Appendix — (DOCX) [file pone.0349153.s002.docx]

| Participant ID Number |  |
| --- | --- |
| Date |  |
| Visit code |  |
| Interview site | 1, Dandora 2 \| 77, Other, specify |
| Other, please specify | (text) |
| **Introduction and lead education.** | Lead education (Please cover the following key points with the participant) |
| This survey is administered to all participants at their entry visit (Aim 1 (Pilot) participants and Aim 2 (RCT) participants). | Lead is a common toxic metal used in some workplaces and industries and is a contaminant in some products such as paint or recycled metal pots.  Lead may enter a child’s body if they breathe in or eat lead-containing air, dust, soil, food or materials.  Inside the body, lead is very toxic to children and can cause problems in growth and development.  Too much lead in the body may affect the child’s growing brain and contribute to learning problems or behavioral problems.  The best way to know if your child has lead in their body is to test for lead in their blood.  The best treatment and prevention of lead problems for children is to remove any ongoing sources from the child’s environment.    This survey contains questions in four sections to help you identify potential sources of lead for your child. It will take about ten minutes of your time to answer them.  At the end, we will discuss protective actions you can take. |
| **Section 1. Time at home** |  |
| 1. 1. How long has your child lived in their current house/home? | [months] (text) |
| 1. 2. Where does (child’s name) spend most of the time during a typical day? Please select one. | 1, In the home or around the home compound \|  2, At school, please specify \| 3, Somewhere else in the community, please specify \| 99, Don’t know |
| 3. If at school, please specify the school: | (text) |
| 4. If somewhere else, please specify: | (text) |
| **Section 2. Potential sources near the home** | The following questions are about potential sources near the child’s home. The history of lead added to petrol and paint or nearby lead related industrial activities may contribute to lead being in soil or air near your home. |
| 1. Does the child’s home have paint on the exterior surfaces such as the walls or windowsills or door(s)? | 1, Yes \| 0, No \| 99, Don’t know |
| 2. If yes, is any of the exterior surface paint chipping or peeling? | 1, Yes \| 0, No \| 99, Don’t know |
| Does the child play or spend time in any of the following areas? (Answer yes, no or don’t know for each) |  |
| 3. Bare soil/dirt around the home compound | 1, Yes \| 0, No \| 99, Don’t know |
| 4. Bare soil/dirt at nearby neighborhood play areas | 1, Yes \| 0, No \| 99, Don’t know |
| 5. Near the Dandora dumpsite (within 20 meters) | 1, Yes \| 0, No \| 99, Don’t know |
| 6. Near other dumpsite (within 20 meters) | 1, Yes \| 0, No \| 99, Don’t know |
| Is the child’s home/compound within 20 meters of any of the following? (Answer yes, no or don’t know for each) |  |
| 7. Busy road | 1, Yes \| 0, No \| 99, Don’t know |
| 8. Near rubbish burning site | 1, Yes \| 0, No \| 99, Don’t know |
| 9. Waste recycling activity or shop | 1, Yes \| 0, No \| 99, Don’t know |
| 10. Construction activities that produce dust | 1, Yes \| 0, No \| 99, Don’t know |
| 11. Garage activity (auto repair, radiator repair) | 1, Yes \| 0, No \| 99, Don’t know |
| 12. Heating or melting lead activity such as soldering pipes, welding, torch work, metal jewelry making | 1, Yes \| 0, No \| 99, Don’t know |
| 13. Painting work (applying or removal of paint from cars, bicycles, furnishings/ furniture) | 1, Yes \| 0, No \| 99, Don’t know |
| 14. Battery recycling or repair | 1, Yes \| 0, No \| 99, Don’t know |
| 15. Ceramic glazing activity | 1, Yes \| 0, No \| 99, Don’t know |
| 16. Any other nearby (in home or on compound) activities that may involve lead? | 1, Yes \| 0, No \| 99, Don’t know |
| 17. Describe other nearby lead activities, if yes: | (text) |
| **Section 3. Potential sources inside the home** | Some household products and materials inside the home may contain lead. The following questions are about potential sources inside the child’s home**.** |
| 1. Do household members remove outdoor shoes before entering the house? | 1, Yes \| 0, No \| 99, Don’t know |
| 2. Do you have a mat at the entrance to wipe/ dust shoes/feet before entering the house? | 1, Yes \| 0, No \| 99, Don’t know |
| 3. Does the child’s home have interior painted surfaces such as wall(s), windowsills, door frames, or the floor? | 1, Yes \| 0, No \| 99, Don’t know |
| 4. If yes, is the paint on any of these surfaces peeling or chipping? | 1, Yes \| 0, No \| 99, Don’t know |
| 5. Does the home contain furnishings that are painted such as tables, chair, or bed? | 1, Yes \| 0, No \| 99, Don’t know |
| 6. If yes, is the paint on any of these furnishings peeling or chipping? | 1, Yes \| 0, No \| 99, Don’t know |
| 7. Does this home have a dirt/ soil floor? | 1, Yes \| 0, No \| 99, Don’t know |
| 8. Does this home have a carpet or carpets on the floor? | 1, Yes \| 0, No \| 99, Don’t know |
| How are the floors cleaned in this house? (Answer yes, no, or don’t know for each): |  |
| 9. Broom | 1, Yes \| 0, No \| 99, Don’t know |
| 10. Wet mop or wet rug | 1, Yes \| 0, No \| 99, Don’t know |
| 11. Other cleaning method, please specify: | 1, Yes \| 0, No |
| 12. If other cleaning method, please specify: (text) | (text) |
| 13. Which is the most used source of drinking water? (Tick the most common one only) | 1, Piped water in the house \| 2, Piped water outside the house (shared) \| 3, Water vendor \| 4, Borehole \| 5, Rainwater \| 77, Other, please specify: |
| 14. If other source of drinking water, please specify: (text) | (text) |
| 15. Which is the most used source of cooking water? (Tick the most common one only) | 1, Piped water in the house \| 2, Piped water outside the house (shared) \| 3, Water Vendor \| 4, Borehole \| 5, Rainwater \| 77, Other, please specify: |
| 16. Other cooking water source, please specify (text) | (text) |
| 17. Does the household store their drinking water? | 1, Yes, in plastic container \| 2, Yes, in metal container \| 3, Yes, in glazed clay pot \| 4, Yes, in glass container \| 5, No, the household does not store drinking water \| 99, Don’t know \| 77, Other type of storage container, please specify |
| 18. Other water storage, please specify: | (text) |
| 19. Is there paint on the inside of any of the water storage containers? | 1, Yes \| 0, No \| 99, Don’t know |
| **Section 4. Products that may contain lead** | The next questions are about products that you may use on your child that could contain lead. |
| Are any of the following cosmetics/makeup or medicines *used on the child*? (Answer yes, no, or don’t know to each) |  |
| 1. Wanja/Kohl/surma/kajal | 1, Yes \| 0, No \| 99, Don’t know |
| 2. Sindoor | 1, Yes \| 0, No \| 99, Don’t know |
| 3. Turmeric applied to skin (soap, cream, oil) | 1, Yes \| 0, No \| 99, Don’t know |
| 4. Have you ever given your child herbal remedies? | 1, Yes \| 0, No \| 99, Don’t know |
| 5. When was the last time you gave your child herbal remedies? | 1, More than a year ago \| 2, Less than a year ago \| 3, Less than 30 days ago \| 99, Don’t know \| 77, Other, specify |
| 6. Other time gave child remedies, please specify | (text) |
| 7. Where do you get the herbal / traditional remedies? | 1, Shop \| 2, Market \| 3, Herbalist \| 4, Traditional medicine man/woman \| 77, Other, specify |
| 8. Other source for remedies, please specify | (text) |
| **Section 5. Diet and feeding** | The next questions are about feeding your child |
| 1. Where do you get your vegetables and fruits from (tick all that apply) | 1, I grow them in the home garden \| 2, From local market \| 3, Buy from my neighbor who grows them |
| 2. What type of cooking pots do you use in the house?. (Tick all that apply)(see job aid with pictures) | 1, Branded metal sufuria \| 2, Unbranded metal sufuria \| 3, Jua-kali sufuria \| 4, Teflon (non-stick) coated pot \| 5, Clay cooking pot \| 6, Ceramic glazed pot \| 77, other specify |
| 3. Other type of cooking pot, specify | (text) |
| 4. (Tick all that apply) What type of plate or bowl do you use most of the time when feeding your child? | 1, Glazed ceramic dish, bowl or jug \| 2, Metal dish bowl or jug \| 3, Plastic dish, bowl or jug \| 4, Melamine \| 77, Other, describe |
| 5. Other type of plate/bowl, describe | (text) |
| 6. What type of cup do you use? | 1, Glazed ceramic cup or jug \| 2, Metal cup or jug \| 3, Plastic cup or jug \| 4, Melamine \| 77, Other, describe |
| 7. Other cup, describe | (text) |
| 8. What kind of bottle do you use for giving child water or juice? | 1, Glazed ceramic \| 2, Metallic \| 3, Plastic \| 4, Melamine \| 77, Other, describe |
| 9. Other bottle, describe | (text) |
| 10. Do you add any spices to child’s food?  If yes, then proceed… | 1, Yes \| 0, No |
| 11. Pilau mix/masala | 1, Yes \| 0, No |
| 12. Curry powder | 1, Yes \| 0, No |
| 13. Turmeric | 1, Yes \| 0, No |
| 14. Chili powder | 1, Yes \| 0, No |
| 15. Coriander powder | 1, Yes \| 0, No |
| 16. Cinnamon | 1, Yes \| 0, No |
| 17. Mixed spices /sambar masala/garam masala | 1, Yes \| 0, No |
| For spices that you purchase, where are they purchased? (tick all that apply) |  |
| 18. Loose spices from local markets | 1, Yes \| 0, No \| 99, Don’t know |
| 19. Supermarkets/shops | 1, Yes \| 0, No \| 99, Don’t know |
| 20. Packed spiced sold by the local brands | 1, Yes \| 0, No \| 99, Don’t know |
| 21. Other place spices purchased, please specify: (text) | (text) |
| 22. How many days in the past seven days did your child consume green leafy vegetables such as Sukuma wiki, spinach, broccoli, amaranth, or other dark green leaves? | Number of days in past 7 days: |
| 23. What was the main source of the dark green leafy vegetables? | 1, Grown in the compound \| 2, Purchased from local open outdoor market \| 3, Purchased from local supermarket/grocery store/similar indoor shop \| 4, Purchased from or eaten in a restaurant or food vendor \| 77, other |
| 24. If other green leafy vegetable source, please specify: (text) | (text) |
| 25. In the last 12 months, was your child given iron supplements/ tablets or syrup? | 1, Yes \| 0, No \| 99, Don’t know |
| **Section 6. Household member work activities** | The next questions are about work activities of your household members. If they are involved in activities with lead, it is possible they could bring lead home as dust on their shoes and clothing. |
| *For each “yes” response, capture for whom response is for.*  In the last 6 months, have any members of the child’s household worked in an occupation where they are exposed to lead dust or fumes in a job that may involve lead? Some examples of jobs that may involve lead include: |  |
| 1. Paint work (painting or removing paint such as sandblasting, scraping, sanding, or using a heat gun or torch) | 1, Yes \| 0, No \| 99, Don’t know |
| 2. If yes to paint work, specify whom: | 1, Father \| 2, Mother \| 3, Other relative, please specify \| 88, NA |
| 3. Other relative who does paint work: | (text) |
| 4. Construction work such as demolition or building structures, remodeling, repairing or renovating houses or buildings | 1, Yes \| 0, No \| 99, Don’t know |
| 5. If yes to construction work, specify whom: | 1, Father \| 2, Mother \| 3, Other relative, please specify \| 88, NA |
| 6. Other relative who does construction: | (text) |
| 7. Auto/garage work such as repairing/rebuilding engines or radiators | 1, Yes \| 0, No \| 99, Don’t know |
| 8. If yes to auto/garage work, specify whom: | 1, Father \| 2, Mother \| 3, Other relative, please specify \| 88, NA |
| 9. Other relative who does auto/garage work: | (text) |
| 10. Metal work such as melting metal for reuse (smelting) or metal soldering, melting, foundry or torch work | 1, Yes \| 0, No \| 99, Don’t know |
| 11. If yes to metal work, specify whom: | 1, Father \| 2, Mother \| 3, Other relative, please specify \| 88, NA |
| 12. Other relative who does metal work: | (text) |
| 13. Salvaging metal or scrapyard or dumpsite work | 1, Yes \| 0, No \| 99, Don’t know |
| 14. If yes to salvaging, specify whom: | 1, Father \| 2, mother \| 3, other relative, please specify \| 88, NA |
| 15. Other relative who does salvaging: | (text) |
| 16. Making pottery/ceramics | 1, Yes \| 0, No \| 99, Don’t know |
| 17. If yes to pottery, specify whom: | 1, Father \| 2, mother \| 3, other relative, please specify \| 88, NA |
| 18. Other relative who does pottery: | (text) |
| 19. Working where batteries are manufactured, recycled, repaired or reconditioned | 1, Yes \| 0, No \| 99, Don’t know |
| 20. If yes to batteries, specify whom: | 1, Father \| 2, mother \| 3, other relative, please specify \| 88, NA |
| 21. Other relative who works with batteries: | (text) |
| If yes to any activities above, please answer the following 2 questions |  |
| 22. Do these household/family members wear their work clothes at home or bring their work clothes home? | 1, Always \| 2, Sometimes \| 3, Never \| 99, Don’t know |
| 23. Are their work clothes washed at home? | 1, Always \| 2, Sometimes \| 3, Never \| 99, Don’t know |
| **Section 7. Child Behavior and Health** | The next questions are about child behaviors that influence whether lead gets into the child’s body |
| Mouthing or teething on non-food items is normal in young children; does your child do any of the following? (Answer yes, no, or don’t know for each) |  |
| 1. Put painted objects in mouth | 1, Yes \| 0, No \| 99, Don’t know |
| 2. Put metal objects in mouth | 1, Yes \| 0, No \| 99, Don’t know |
| 3. Eat/chew paint chips | 1, Yes \| 0, No \| 99, Don’t know |
| 4. Eat soil | 1, Yes \| 0, No \| 99, Don’t know |
| 5. Mouth or teeth any other non-food items | 1, Yes \| 0, No \| 99, Don’t know |
| 6. If yes other non-food items, please specify | (text) |
| 7. Does the child wash hands before eating? | 1, Never or rarely \| 2, Sometimes \| 3, Usually \| 4, Always \| 99, Don’t know |
| Does your child get sick frequently? |  |
| 8. Vomiting | 1, Yes \| 0, No \| 99, Don’t know |
| 9.Tummy problems | 1, Yes \| 0, No \| 99, Don’t know |
| 10. Hard stool | 1, Yes \| 0, No \| 99, Don’t know |
| 11. Lack of energy | 1, Yes \| 0, No \| 99, Don’t know |
| 12. Weakness | 1, Yes \| 0, No \| 99, Don’t know |
| 13. Not gaining weight well. | 1, Yes \| 0, No \| 99, Don’t know |
| Does your child have any of the below behavioral problems? |  |
| 14. Hates sitting still | 1, Yes \| 0, No \| 99, Don’t know |
| 15. Difficulty paying attention | 1, Yes \| 0, No \| 99, Don’t know |
| 16. Fights a lot with other children | 1, Yes \| 0, No \| 99, Don’t know |
| 17. Poor school performance | 1, Yes \| 0, No \| 99, Don’t know |
| 18. Slow to talk | 1, Yes \| 0, No \| 99, Don’t know |
| 19. Hearing loss | 1, Yes \| 0, No \| 99, Don’t know |
| 20. Seizures and convulsions | 1, Yes \| 0, No \| 99, Don’t know |
| 21. Comments: | (text) |

| **Section 8. Lead exposure reduction messaging for all participants** |  |
| --- | --- |
| Introduction | Thank you for completing the survey of lead risk factors. Now I would like to offer you some guidance on how to avoid exposing your child to metal toxins like lead. (Provide the participant with the exposure reduction hand-out, review the pictures as you provide the messages below) |
| Lead & child health messaging: | As we discussed at the beginning of the survey:  Lead is a toxic metal.  Lead is particularly dangerous to young children.  It can influence their growth and development. Children’s brain development is very sensitive to lead. Too much lead exposure may affect the child’s growing brain and contribute to learning problems or behavioral problems.  The best way to know if your child is exposed to too much lead is a blood lead test.  The best treatment for and prevention of too much lead exposure is to remove any ongoing sources. |
| Dietary messaging:  Staff – please review the dietary guidance component of the factsheet for all participants at their study entry visit | Did you know that a healthy diet can help protect a child from lead? Providing foods that contain vitamin C, iron and calcium in your child’s diet can help.  This handout shows some of the foods that are good sources of these vitamins for your child. Include these foods as part of your child’s regular diet. |
| 1. Dietary messages were discussed | 1, Yes \| 0, No |
| Everyday habits messaging:  Staff – please review the everyday habits to reduce your child’s exposure to lead component of the factsheet for all participants. | Did you know that lead can sometimes be found in house dust, paint chips, and soil? This handout shows some everyday habits that can reduce your child’s exposure to lead in soil or house dust.  Wash your child’s hands with soap and water before meals and when returning from outdoor play  Control dust build up on floors and surfaces with frequent cleaning of floors and surfaces in your home with soap and water.  Do not allow your child to eat soil or paint chips.  Do not allow household members to wear outdoor shoes in the home.  Discourage child play in bare soil areas. Choose areas with grass or other coverings. |
| 2. Everyday habits messages discussed. | 1, Yes \| 0, No |
| Staff – please review only the specific potential sources or risk factors below that were noted on this participant’s survey. Use the accompanying guidance as applicable. Use the handout to show the related pictures. | There are some specific additional sources of lead that may be important for your child based on your helpful response to the survey questions. Let’s review those. |

| **Section 9. Tailored messaging per survey results** | **Action steps** |
| --- | --- |
| Potential sources of risk factors identified for this child from survey response: | Recommended messaging for exposure reduction |
| This child’s home has a **dirt/mud/soil floor.** | Covering the floor with a surface that can be routinely washed with soap and water is advised. |
| 1. This risk factor (flooring) was identified on the survey. | 1, Yes \| 0, No |
| 2. Messaging on flooring was provided. | 1, Yes \| 0, No |
| This child’s home has **carpet(s)** on the floor | Wash the floor rugs in your home with soap and water regularly to avoid build up of dust and dirt. Hang dry, do not dry by spreading out on soil or the ground. |
| 3. This risk factor (carpeting) was identified on the survey. | 1, Yes \| 0, No |
| 4. Messaging on carpeting was provided. | 1, Yes \| 0, No |
| This home is reported to have **chipping or peeling paint in the home on surfaces or products.** | It’s best to cover any peeling or chipping painted surfaces with fresh paint. If it is a small area, consider covering it with thick tape or taping paper over it. Remove any child toys or furnishings with chipping/peeling paint. Don’t allow the child to mouth or chew on any painted surfaces with chipping or peeling paint |
| 5. This risk factor (peeling paint) was identified on the survey. | 1, Yes \| 0, No |
| 6. Messaging on peeling paint was provided. | 1, Yes \| 0, No |
| This child’s home reports using **cookware or dishware/ utensils that may contain lead** (locally made or artisanal recycled aluminum cookware or dishware, glazed ceramic pot or dishware) | It's important to replace the cookware and dishes that may contain lead when preparing food or serving food for your child. Avoid recycled/scrap aluminum, locally made recycled aluminum pots, sufuria or glazed ceramic pots or dishware. Safer alternatives include stainless steel, plastic, glass and non-glazed ceramic or cast iron. |
| 7. This risk factor (cookware) was identified on the survey. | 1, Yes \| 0, No |
| 8. Messaging on (cookware) was provided. | 1, Yes \| 0, No |
| This child eats foods grown on the compound (shamba/ kitchen garden) | It’s important to wash well/ thoroughly crops you grow to remove any dirt particles. Sometimes dirt contains lead. |
| 9. This risk factor (garden) was identified on the survey. | 1, Yes \| 0, No |
| 10. Messaging on garden food was provided. | 1, Yes \| 0, No |
| The survey indicates that this child uses make up or medicinal products that may contain lead | Some makeup and medicinal products have been found to contain lead. These include some of the products you mentioned (remind the participant). They are not recommended for use on children. |
| 11. This risk factor (make up/medicinal) was identified on the survey. | 1, Yes \| 0, No |
| 12. Messaging on makeup/medicinal was provided. | 1, Yes \| 0, No |
| This survey indicates that the child uses traditional medicines or remedies. | Some traditional medications or remedies may contain lead. We recommend that children avoid them. |
| 13.This risk factor (medicine/remedy) was identified on the survey. | 1, Yes \| 0, No |
| 14. Messaging on medicines/remedy) was provided. | 1, Yes \| 0, No |
| This child’s foods are commonly prepared with spices that have been found in some cases to contain lead. | Sometimes, loose spices that are sold without any branding, such as the KEBS mark of standardization, may contain lead. It is best to purchase packed spices from local brands or branded spices available in supermarkets or shops. |
| 15. This risk factor (spices) was identified on the survey. | 1, Yes \| 0, No |
| 16. Messaging on spices was provided. | 1, Yes \| 0, No |
| This child’s survey identified some potential activities nearby that may put lead into the air and that can settle onto surfaces including dirt. (Staff: review the nearby sources identified on the survey) | Community activities that may produce lead should be areas to avoid for your child’s outdoor time. Avoid your child playing or spending time near these areas. |
| 17. This risk factor (activities) was identified on the survey. | 1, Yes \| 0, No |
| 18. Messaging on activities was provided. | 1, Yes \| 0, No |
| This child’s household includes members with potential workplace exposure to lead. (Staff: review the specific lead related work activities for household members) | Some jobs that involve lead can leave lead dust on workers' clothing and shoes. It is important to ensure that household members that work with lead don’t bring contaminated lead clothes and shoes in their homes. It is advised for workers who encounter lead on the job leave their work shoes outside of the home, wash their work clothing separately from child’s clothing, and remove work clothing before interacting with the child. |
| 19. This risk factor (workplace exposure) was identified on the survey. | 1, Yes \| 0, No |
| 20. Messaging on workplace exposure was provided. | 1, Yes \| 0, No |
| This child is reported to mouth or chew on non-food objects. | Children who put objects in their mouth may ingest lead from those objects. Wash any object that your child regularly mouths regularly with soap and water. If the object is metal or painted, remove it from your home or the child’s access to it. |
| 21. This risk factor (mouthing) was identified on the survey. | 1, Yes \| 0, No |
| 22. Messaging on mouthing was provided. | 1, Yes \| 0, No |
| Staff person completing this form |  |
| Form completion time |  |
