# Supplementary material for: Adapting, piloting, and evaluating a pediatric lead screening and risk-reduction intervention in Nairobi: A hybrid implementation-effectiveness trial protocol
Source: PLoS One. 2026 Jun 4;21(6):e0349153. doi: 10.1371/journal.pone.0349153 (PMC13235933; doi:10.1371/journal.pone.0349153)
Supplement: S3 Appendix — (DOCX) [file pone.0349153.s003.docx]

Title: Lead Exposure Intervention Program (LEIP)

**STUDY PROTOCOL**

Version Number: 2.0

January 2026

**PROTOCOL PRINCIPAL INVESTIGATORS**

**MPI:** Elizabeth Maleche-Obimbo (University of Nairobi)

**MPI:** Sarah Benki-Nugent (University of Washington)

**MPI:** Catherine Karr (University of Washington)

KEY ROLES & CONTACT INFORMATION

| **MULTI-PRINCIPAL INVESTIGATORS / PROJECT Directors** |  |  |  |
| --- | --- | --- | --- |
| **Elizabeth Maleche Obimbo,** MBChB, MMed, MPH, FPulm, PhD,  Professor, Department of Paediatrics & Child Health, University of Nairobi.  PO Box 19676-00202, Nairobi, Kenya  <Tel:254-20-4915046>, Email:[elizabeth.obimbo@uonbi.ac.ke](mailto:elizabeth.obimbo@uonbi.ac.ke); lisaobimbo@gmail.com;  **Sarah Benki-Nugent**, MS, PhD, Associate Professor,  Div. of Allergy and Infectious Diseases, Global Health, University of Washington,  Hans Rosling Center 888, 3980 15th Ave., Box 351620, Seattle, WA 98195  Tel: 206-550-0758, Email: benki@uw.edu | | | |
| **Catherine Karr**, MD PhD, Professor,  Dept. of Pediatrics, Div. of General Pediatrics, Dept. of Environmental & Occupational Health Sciences, University of Washington,  4245 Roosevelt Way NE, Suite 100, Seattle, WA 98105  Email: ckarr@uw.edu  **CO-INVESTIGATORS** | | | |
| **Beatrice Mutai,** Lecturer, MBChB, MMed.  Department of Paediatrics & Child Health, University of Nairobi,  P.O. Box 19676-00202, Nairobi-Kenya  Tel: 254-708-552-909, Email: bmutai@uonbi.ac.ke; [mutaibc@gmail.com](mailto:mutaibc@gmail.com) | | | |
| **Farida H. Were, BSc, MSc,** PhD, Lecturer  Department of Chemistry, University of Nairobi,  P.O. Box 30197-00100, Nairobi-Kenya  <Tel:+254-729-239-135>,Email: [fwere@uonbi.ac.ke](mailto:fwere@uonbi.ac.ke); [faridares@yahoo.com](mailto:faridares@yahoo.com); | | | |
| **Ferdinand Mukumbang**, MPhil, PhD, Assistant Professor,  Department of Global Health, University of Washington, Hans Rosling Center 888, 3980 15th Ave., Box 351620, Seattle, WA 98195  Tel: (206) 496-5397, Email: [ferdie@uw.edu](mailto:ferdie@uw.edu) | | | |
| **Barbra Richardson**, MS, PhD, Research Professor,  Department of Biostatistics, University of Washington,  3980 15th Ave. NE, Seattle, WA, 98195  Tel : 206-550-7540, Email: [barbrar@uw.edu](mailto:barbrar@uw.edu) | | | |
| **Cyrus Mugo**, MBChB, MPH, PhD, Reasearch Scientist,  Department of Research and Programs, Kenyatta National Hospital, PO Box 20723-00202 Hospital Road, Upper Hill, Nairobi, Kenya  Tel: Tel: +254 721 599 626  Email: cyrusmugodr@gmail.com | | |  |
| **Anne Riederer,** ScD, Affiliate Associate Professor,  Department of Environmental and Occupational Health Sciences, University of Washington.  1959 NE Pacific Street, Box 357234, Seattle, WA 98195  Tel: 404-805-4982, Email: [anneried@uw.edu](mailto:anneried@uw.edu) | |  |  |

COLLABORATING INSTITUTIONS AND FUNDING

This study will be conducted in collaboration with Kenyatta National Hospital (KNH), the University of Nairobi, and the University of Washington.

| **Institutions** | University of Nairobi  P.O. Box 30197-00101  Nairobi, Kenya |
| --- | --- |
|  | Kenyatta National Hospital  PO Box 20723-00202  Hospital Road, Upper Hill  Nairobi 00202, Kenya |
|  | University of Washington  4333 Brooklyn Avenue NE, Box 359472  Seattle, WA 98195 USA |
| **FUNDING AGENCY**  Funding type: Grant Award No. R01ES036010  Name of Funding Source: National Institute on Environmental Health Sciences (NIEHS)  Principal Investigators on Proposal: Catherine Karr, MD, PhD, Elizabeth Maleche-Obimbo, MBChB, MMed, MPH, PhD. Sarah Benki-Nugent, MS, PhD  Title of Proposal: Health Cities for Healthy Brains: Implementation of a Lead Exposure Intervention Program in Nairobi (LEIP)  Dates: 25 April 2025 to 24 April 2030 | |

LIST OF ABBREVIATIONS & ACRONYMS

| ABC | Air Pollutant Exposures and Brain Development in Children |
| --- | --- |
| BLL | Blood Lead Level |
| CITI | Collaborative Institutional Training Initiative |
| CFIR | Consolidated Framework for Implementation Research constructs |
| CFR | Code of Federal Regulations |
| ERC | Ethics and Research Committee |
| FDA | Food and Drug Administration |
| HIC | High-Income Country |
| HSD | Human Subjects Division |
| IDI | In-depth Interview |
| ICH | International Council for Harmonization (Good Clinical Practice) |
| ICP-MS | Inductively coupled plasma mass spectrometry |
| IRB | Institutional Review Board |
| KAP | Knowledge, Attitudes and Practices |
| KII | Key Informant Interview |
| KNH | Kenyatta National Hospital |
| LEIP | Lead Exposure Intervention Program |
| LMIC | Low- and Middle-Income Country |
| MA | Massachusetts |
| ml | milliliter |
| NIH | National Institute of Health |
| POC | Point of Care |
| RCT | Randomized Control Trial |
| RANAS | Risks, Attitudes, Norms, Abilities, and Self-regulation (framework) |
| SMS | Short Message Service |
| SSA | Sub-Saharan Africa |
| ug/dL | Microgram per Deciliter |
| ULAB | Used Lead-Acid Batteries |
| UNICEF | United Nations Children’s Fund |
| UoN | University of Nairobi |
| UW | University of Washington |
| US/USA | Unites States |
| USD | Unites States Dollar |
| WHO | World Health Organization |
| XRF | X-ray Fluorescence |

OPERATIONAL DEFINITIONS

Normal blood lead level = lead concentration < 5 micrograms per deciliter.

High blood lead level = blood lead concentration ≥ 5 micrograms per deciliter.

STATEMENT OF COMPLIANCE

The study will be conducted in accordance with the International Conference on Harmonization guidelines for Good Clinical Practice (ICH E6), the Helsinki Declaration and Code of Federal Regulations on the Protection of Human Subjects (45 CFR Part 46), and the Terms of Award. All personnel involved in the conduct of this study have completed human subjects' protection training.

TABLE OF CONTENTS

**Page**

[KEY ROLES & CONTACT INFORMATION ii](#_Toc227653977)

[COLLABORATING INSTITUTIONS AND FUNDING iv](#_Toc227653978)

[LIST OF ABBREVIATIONS & ACRONYMS v](#_Toc227653979)

[OPERATIONAL DEFINITIONS vi](#_Toc227653980)

[STATEMENT OF COMPLIANCE vii](#_Toc227653981)

[TABLE OF CONTENTS viii](#_Toc227653982)

[STRUCTURED SUMMARY x](#_Toc227653983)

[INTRODUCTION 12](#_Toc227653984)

[LITERATURE REVIEW 13](#_Toc227653985)

[RATIONALE 15](#_Toc227653986)

[STUDY QUESTIONS AND HYPOTHESES 16](#_Toc227653987)

[GENERAL OBJECTIVE 17](#_Toc227653988)

[METHODS 18](#_Toc227653989)

[**Study Design** 18](#_Toc227653990)

[AIM 1 19](#_Toc227653991)

[**Aim 1. Overview** 19](#_Toc227653992)

[**Aim 1. Study Area Description and Study Site** 19](#_Toc227653993)

[**Aim 1. Study Population** 19](#_Toc227653994)

[*Inclusion Criteria* 19](#_Toc227653995)

[*Exclusion Criteria* 19](#_Toc227653996)

[**Aim 1. Sample Size and Rationale** 19](#_Toc227653997)

[**Aim 1. Screening and Selection of Study Participants** 20](#_Toc227653998)

[**Aim 1. Recruitment and Consenting Procedures** 20](#_Toc227653999)

[**Aim 1. Data Collection Procedures** 21](#_Toc227654000)

[*Aim 1 Enrollment Visit:* 21](#_Toc227654001)

[*Aim 1 Follow-up Procedures:* 23](#_Toc227654002)

[AIM 2 25](#_Toc227654003)

[**Aim 2. Overview** 25](#_Toc227654004)

[**Aim 2. Study Area Description and Study Site** 25](#_Toc227654005)

[**Aim 2. Study Population** 25](#_Toc227654006)

[*Inclusion criteria* 25](#_Toc227654007)

[*Exclusion criteria* 25](#_Toc227654008)

[**Aim 2 Sample Size and Power** 25](#_Toc227654009)

[**Aim 2. Screening and Selection of Study Participants** 26](#_Toc227654010)

[**Aim 2. Recruitment and Consenting Procedures** 26](#_Toc227654011)

[**Aim 2. Data Collection Procedures** 26](#_Toc227654012)

[*Aim 2 Enrollment Visit:* 27](#_Toc227654013)

[*Aim 2 Follow-up Procedures (randomized participants only):* 28](#_Toc227654014)

[AIM 3 29](#_Toc227654015)

[**Aim 3. Overview** 29](#_Toc227654016)

[**Aim 3. Study Area Description and Study Site** 29](#_Toc227654017)

[**Aim 3. Study Population** 29](#_Toc227654018)

[**Aim 3. Sample Size and Power** 30](#_Toc227654019)

[**Aim 3. Screening and Selection of Study Participants** 30](#_Toc227654020)

[**Aim 3. Recruitment and Consenting Procedures** 30](#_Toc227654021)

[**Aim 3. Data Collection Procedures** 31](#_Toc227654022)

[VARIABLES FOR AIMS 1, 2, and 3 32](#_Toc227654023)

[**Referrals for Children with Concern for Need of Chelation** 35](#_Toc227654024)

[**Participant’s Results Sharing** 35](#_Toc227654025)

[**Instruments, Materials, Supplies and Laboratory Procedures** 35](#_Toc227654026)

[**Training Procedures** 36](#_Toc227654027)

[**Quality Assurance Procedures** 36](#_Toc227654028)

[ETHICAL CONSIDERATIONS 38](#_Toc227654029)

[**Ethical Standard** 38](#_Toc227654030)

[**Institutional Review Board** 38](#_Toc227654031)

[**Potential Risks and Benefits** 38](#_Toc227654032)

[POTENTIAL BENEFITS 40](#_Toc227654033)

[**Informed Consent Process** 40](#_Toc227654034)

[PARTICIPANT CONFIDENTIALITY AND PRIVACY 42](#_Toc227654035)

[**Future Use of Stored Specimens and Other Identifiable Data** 42](#_Toc227654036)

[**Retention Plan** 42](#_Toc227654037)

[DATA MANAGEMENT 44](#_Toc227654038)

[**Types of Data** 44](#_Toc227654039)

[**Data Entry and Cleaning** 44](#_Toc227654040)

[**Data Storage** 44](#_Toc227654041)

[**Study Records Retention** 44](#_Toc227654042)

[ANALYSIS PLAN 45](#_Toc227654043)

[STUDY RESULTS DISSEMINATION PLAN 48](#_Toc227654044)

[ITEMIZED BUDGET 49](#_Toc227654045)

[STUDY TIMELINE 50](#_Toc227654046)

[STUDY LIMITATIONS 51](#_Toc227654047)

[REFERENCES 52](#_Toc227654048)

STRUCTURED SUMMARY

| **Title:** | Lead Exposure Intervention Program (LEIP) |
| --- | --- |
| **Objective:** | This project seeks to adapt pediatric blood lead level testing and “healthy home” lead prevention program experience in the U.S. for a sub-Saharan Africa (SSA) city context - where blood lead testing and follow up protocols are needed, The project will pilot and evaluate strategies for delivery of messages to parents on ways to reduce their child’s lead exposure. |
| **Aims:** | **Aim 1**. **Adapt, pilot and refine a prototype child lead exposure risk survey and messaging protocol**  **Aim 2.** **Evaluate uptake of tailored risk reduction** **messaging delivered in clinic vs at home**  **Aim 3**. **Evaluate barriers at individual and structural levels** |
| **Methods:** | Design:  Aim 1 will consist of one-arm pilot of a lead Lead Risk Survey and risk reduction messaging. We will leverage a recent young child cohort in Nairobi (ABC; P22/01/202; N=350; anticipated child age 36 to 48 months), to examine in-clinic survey accuracy and the understandability and acceptability of risk reduction messaging using concurrent home observation and cognitive interviews in a subset with high child blood lead level (BLL). We will use exit in-depth interviews (IDIs) to further refine the survey and risk reduction messaging.  Aim 2 will consist of a randomized trial. Parent-child dyads (child age 12 to 72 months) attending routine well-child visits will receive lead point-of-care testing and complete the Lead Risk Survey (up to N=1,500). Those with high BLL (N=100) will be randomized to 1) immediate in-clinic messaging tailored per their survey (n=50) or 2) immediate in-clinic messaging and addition of home observation and reinforced messages tailored per home findings (n=50).  Aim 3 will consist of a qualitative study. Exit in-depth interviews (IDIs) administered to parents of children with high BLL will be used to identify individual level barriers to uptake of risk reduction measures. Key informant interviews (KIIs) with key informants will be used to identify broader structural barriers to inform policy. |
| **Population:** | Parent-child dyads in Nairobi, Kenya |
| **Sites:** | Dandora II Health Centre, Baba Ndogo Health Centre, Kariokor Health Centre, Kariobangi Health Centre, and Eastlands Health Centre |
| **Study Duration:** | May 1, 2025 – April 30, 2030 |
|  |  |
| **Subject Participation Duration:** | Aim 1: 6 months, Aim 2: 9 months, Aim 3: qualitative data collection only |
| **Estimated Time to Complete Enrollment:** | Aim 1: 1 year, Aims 2 and 3: 1 year |
| **Outcomes:** | Aim 1: Acceptability, understandability of lead exposure program protocol, survey self-report vs. at home observation, refined survey instrument and messages.  Aim 2: Recall of messaging, behavior change to reduce exposure, uptake of blood lead level re checks, and child blood lead level change.  Aim 3: Barriers to behavior change, understandability, acceptability and appropriateness of the lead testing program. |

INTRODUCTION

In the U.S., marked reductions in child lead exposure is noted as one of its greatest public health accomplishments. Core features of successful programs in the U.S. include screening to identify children with higher blood lead levels (BLLs), “healthy home” programs to identify household sources, surveillance, and regulatory policy to remove lead from the environment. Yet lead toxicity remains a major global public health concern, accounting for 63% of the global burden of idiopathic intellectual disability. Evidence is clear that child blood lead levels (BLLs) once considered "safe” (< 10 ug/dL) are linked to compromised cognitive and behavioral development. Among the one in three children with BLL exceeding current WHO guidelines (≥ 5 ug/dL), 90% reside in low and middle income countries (LMICs). Both WHO and UNICEF highlight the need for programs to identify children with higher BLLs in LMICs, including sub-Saharan Africa (SSA), and need for protocols to respond to cases of elevated exposure. In the following protocol, we propose to adapt BLL screening and “healthy home” lead prevention program experience in the U.S. for an SSA city context - where BLL testing and follow up protocols are needed most and can leverage the city context to build awareness - and evaluate them in foundational implementation activities in Nairobi.

LITERATURE REVIEW

We seek to increase awareness of lead exposure and build a foundation for sustained efforts to reduce pediatric lead exposure in LMIC cities by implementing components of well-established lead prevention programs developed in HICs in an urban SSA context. The proposed research will be conducted in Nairobi, Kenya’s capital city of 4,397,073 people at the 2019 census, specifically Dandora and nearby communities.*^[[1]](#endnote-1)^* **Like other urban informal settlement communities,*****^[[2]](#endnote-2),^******^[[3]](#endnote-3)^* Dandora is characterized by high population density, poor infrastructure, and clusters of largely unregulated industries**. It is also home to Nairobi’s principal dumpsite, the 30+ acre Dandora landfill.*^[[4]](#endnote-4)^*

a. Why lead exposure in children matters, particularly in LMICs. **Lead toxicity is a major global public health concern with disproportionate impact for children in SSA**. The WHO defines blood lead levels (BLLs) ≥ 5 ug/dL as high.*^[[5]](#endnote-5)^* An estimated one in three children worldwide, 90% of whom live in LMICs, have BLLs ≥ 5 ug/dL.*^[[6]](#endnote-6)^*  UW’s Institute for Health Metrics and Evaluation estimates that 2,831,801 Kenyan children have BLLs $\geq$ 5 ug/dL, with 326,521 over 10 ug/dL*.^6^* Lead is responsible for estimated 98 million lost IQ points in Africa, a cost of ~ 4% of GDP*^[[7]](#endnote-7)^ --* 2-3 times the cost of pediatric lead exposure in Europe and the USA.*^7^*

Available data demonstrate that BLLs in children living near areas with high lead in soil and house dust in LMICs can be high.*^[[8]](#endnote-8)^* A 2021 systematic review of LMIC data reports pooled median BLLs for children in SSA countries ranging from 5.3 - 8.7 ug/dL (Kenya was not included due to inadequate data availability).*^[[9]](#endnote-9)^* **For BLLs in the range of 5 - 10 ug/dL, overt poisoning signs are not present, however, these exposures have been shown to interrupt healthy brain development, and are clearly associated with cognitive deficits (IQ point loss), behavioral changes such as reduced attention span, increased risk of antisocial and aggressive behavior, and reduced educational succes**.*^[[10]](#endnote-10)^*

b. Why lead in SSA cities is critical to address. Features of SSA urban environments contribute to increased risks of lead exposure. At the same time, these settings present important opportunities, increased access to resources, and economies of scale for addressing this urgent public health issue compared to rural areas.*^2,3^* While adequate data is lacking in SSA, available **evidence suggests that urban pediatric lead exposures typically exceed non-urban settings**. This reflects the density of housing in urban centers, poor housing quality in informal urban settlements, the proximity of housing to busy roadways, the presence of large unregulated dumpsites and open channels of untreated municipal and industrial wastewater, and the concentration of informal and formal industries that emit lead such as used lead acid battery (ULAB) recycling, auto repair shops, and e-waste recycling.*^[[11]](#endnote-11)^*^-^*^,^^[[12]](#endnote-12),^^[[13]](#endnote-13),^^[[14]](#endnote-14),^^[[15]](#endnote-15),^^[[16]](#endnote-16),^^[[17]](#endnote-17),^^[[18]](#endnote-18),^^[[19]](#endnote-19),^^[[20]](#endnote-20),^**^[[21]](#endnote-21),^^[[22]](#endnote-22),^**^[[23]](#endnote-23)^* Lead in urban tap water from older pipes and food grown or raised in contaminated urban soils may also contribute to urban SSA children’s lead exposure.*^[[24]](#endnote-24)^*^-^*^,^^[[25]](#endnote-25),^^[[26]](#endnote-26)^* Products in the home, notably lead paint, lead contaminated cookware and dishes, cosmetics, and toys may be insufficiently regulated.*^[[27]](#endnote-27)^* Existing data on lead sources, exposure routes and BLL distributions in Nairobi and other SSA cities derive from *ad hoc* and small-scale environmental and epidemiological studies. A 2007 study of 387 children age 6-59 months in one of Nairobi’s large informal settlements (Kibera) reported 7% with BLL > 10 ug/dL .*^21^* In Mombasa, Kenya’s second largest city, BLLs were measured in 130 children age 12-59 months using the LeadCare II^TM^ Point of Care (POC) tests in 2014.*^[[28]](#endnote-28)^* Half lived in an informal settlement with a legacy ULAB smelter (closed in 2012) and half lived in an upwind community. More children in the smelter community had BLLs ≥ 10 ug/dL (31% vs. 8%).*^28^* Limited studies from SSA cities suggest contributors to children’s exposures include contaminated soil and house dust, time spent playing outside, and lead paint use (**see** **Preliminary Studies**).

c. Successful programs in HICs have leveraged medical and public health efforts to increase awareness, identify populations at risk and provide blood lead testing for decades. In the USA, policy level efforts have addressed lead in gas, paint, plumbing, food cans, and some infant foods**. These collective actions are touted as one of the 10 greatest public health achievements** in modern U.S. history, as BLLs in American children have declined from a geometric mean of 15 ug/dL in the 1970s to < 1 ug/dL now.*^[[29]](#endnote-29)^*

d. Barriers and opportunities to establish a blood lead screening program in Nairobi. Despite successes of lead programs in HICs, **no established lead screening or surveillance programs currently exist in SSA**, to our knowledge. **Infrastructure needed to support BLL screening includes establishing laboratory capacity as well as medical and public health system expertise**. To date, clinical and commercial laboratories do not routinely offer blood lead testing for children in Kenya. The LeadCare II FDA-approved*^[[30]](#endnote-30)^* POC analyzers (result in 3 minutes) are a practical screening approach employed in studies in Africa and in HIC settings where access to laboratories is a barrier.*^[[31]](#endnote-31)^* In addition, increasing access to portable hand-held direct reading instruments (e.g., X-ray fluorescence [XRF] detectors) to investigate sources in and around a child’s home (soil, dust, paint, cookpots and other products) have become a valuable component of follow up of children with high BLLs in established screening programs.*^[[32]](#endnote-32),^^[[33]](#endnote-33)^* We have established a cadre of UW/US – Nairobi collaborators with relevant expertise and a commitment to advancement of maternal child environmental health in Kenya. This comprises medical and public health, government and academic perspectives.

f. A successful, sustainable lead prevention program in SSA will require multisector, stakeholder engagement and capacity building. Children’s lead exposure has not been a public health political priority in SSA countries, including Kenya,*^[[34]](#endnote-34)^* perhaps in part due to invisibility of exposure, a consequence of lack of lead screening and surveillance. The WHO, UNICEF, USAID, and a recent G-7 workshop highlight importance of lead screening to to address child lead exposure in LMICs, including SSA.*^6,^**^[[35]](#endnote-35)-,^**^[[36]](#endnote-36)^**^[[37]](#endnote-37)^*

RATIONALE

Urban centers such as Nairobi provide a natural opportunity for developing new national capacity for childhood lead exposure prevention programs, with access to existing public health, medical and academic centers and policymakers.

Blood lead screening in children serves two distinct purposes, as demonstrated through well-established programs based in the USA and other HICs (illustrated in Fig. 1). First, screening identifies individual children who are more highly exposed, providing an opportunity to intervene on existing exposures in the child’s environment and prevent continued health impacts (secondary prevention). In addition, community-level BLLs can be used for surveillance at local or national levels, providing powerful data to motivate policy and structural changes, such as regulatory actions, and to showcase evidence of effectiveness of actions taken
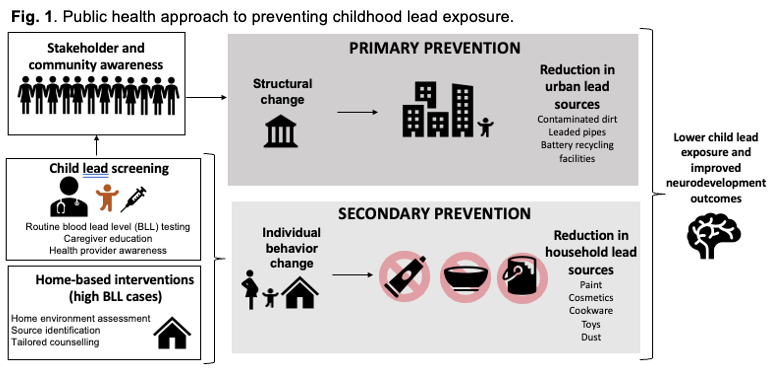
(primary prevention). The novel and foundational activities proposed here respond to the need for addressing child lead exposure in Kenya, leveraging strong preliminary work including identification of concerns from Kenyan stakeholders.

STUDY QUESTIONS AND HYPOTHESES

**Question 1. How can we develop a Lead Risk Survey developed for parents to be understandable, acceptable, and accurate?**

Hypotheses: This is not a hypothesis driven question. We will use parent response to modify/hone the survey for understandability and acceptability. We will compare home observation to clinic based risk reporting to improve the survey.

**Question 2. Do parent participants in an intervention program (LEIP) have good recall of lead exposure source and action steps at 6 and 9 months and does it vary based on how they received messages and the initial severity of the child’s BLL? (primary outcomes) Do participant children return for BLL recheck and is there a change in their BLL at recheck at 9 months? (exploratory outcomes)**

Hypotheses: Recall of risk factors and uptake of messaging will be high in both parents receiving messages in-clinic only and at-home. Messaging uptake will be highest for dyads with higher BLL. More than half of participants will attend follow up blood level rechecks. On average, blood lead levels will decline over the follow up period.

**Question 3. What are the barriers to uptake of lead risk reduction messaging from the perspective of parents participating in LEIP (Aim 2) and key stakeholders?**

Hypotheses. Barriers to risk reduction will include lower household socioeconomic status, lower self-efficacy, and parent occupation in industries involving e-waste, and proximity of housing to lead-contaminated soil.

GENERAL OBJECTIVE

We seek to develop and implement a novel lead testing program in Nairobi.

Specific Aims

Aim 1. Adapt, pilot and refine a prototype child lead exposure risk survey and messaging protocol

Aim 2. Evaluate uptake of tailored risk reduction messaging delivered in clinic vs at home

Aim 3. Evaluate barriers at individual and structural levels

METHODS

## **Study Design**

This study will consist of baseline cross-sectional surveys involving larger sample sizes, with prospective cohort follow-up for a subset of enrolled participants. For Aim 1, an initial pilot survey of a lead exposure risk survey and messaging protocol will be done, and for Aim 2, an implementation randomized trial delivering a lead testing program in two ways (in-clinic only versus clinic-plus at home component). We will conduct in-depth-interviews (IDIs) with a sub-set of parents, and key-informant-interviews (KIIs) of key persons/stakeholders.

The anticipated number of participants for each study aim is shown in Table 1.

| **Table 1: Summary of study aims, study design and sample size for each aim of the LEIP Study** | | | | | |
| --- | --- | --- | --- | --- | --- |
| ***Aim*** | ***Description*** | ***Design*** | ***Population*** | ***Sample size baseline*** | ***Sample size for follow-up cohort subset*** |
| Aim 1 | Pilot study of lead POC testing protocol materials | Cross-sectional baseline  Prospective cohort subset. | Air Pollutant Exposures and Brain Development in Children (ABC) Study participants | 350 parent-child pairs enrolled will have a child lead POC test done | ~20 (all with high BLL) |
| Aim 2 | Implementation trial of lead POC test program in community (clinic only vs with home component) | Cross-sectional baseline  Randomized trial with prospective cohort subset. | Parent child pairs accessing routine well child care at health facilities | 1,500 parent-child pairs enrolled will have a child lead POC test done | 100 children with high BLL (Randomized to 50 per arm) |
| Aim 3 | In-depth-interviews with parents from Aim 2  Key-informant-interviews from key persons / stakeholders | Cross-sectional  (Qualitative) | Parents participating in Aim 2 randomized trial  Key persons /stakeholders | 20 parents for in-depth-interviews (IDI)  20 key persons/ stakeholders for key-informant-interviews (KII) |  |

AIM 1

## **Aim 1. Overview**

Aim 1 will consist of pilot delivery of the LEIP program prototype in the ABC cohort. This will entail a lead POC test, a Lead Risk Survey and messaging. Parent-child pairs with a child found to have high BLL will also be offered a home visit and a lead POC recheck visit.

## **Aim 1. Study Area Description and Study Site**

Recruitment and enrollment will take place at the Dandora II Health Centre. Our current ABC cohort study has a study office at Dandora II Health Centre.

## **Aim 1. Study Population**

Aim 1 will involve parent-child pairs who formerly participated in the Air Pollutant Exposures and Brain Development in Children (ABC) Study.

### *Inclusion Criteria*

- Child was enrolled in ABC study
- Parent or primary caregiver that is a biological relative is available to give informed consent for LEIP study
- Parent/caregiver is 18 years of age or older
- Available for 6 months follow-up
- Willing to allow the collection of blood from the child for a lead POC test

### *Exclusion Criteria*

- Unwilling to be contacted for the purposes of tracing and follow-up

## **Aim 1. Sample Size and Rationale**

A total of 350 parents of children in the ABC cohort will be invited to participate in the lead POC test pilot study.

*Subset for in-depth-interview about the Lead Risk Survey and lead POC test:* Twenty parents will be randomly selected from the 350 enrolled participants to undergo an in-depth-interview to evaluate acceptability and understandability of the Lead Risk Survey.

*Subset for home visit and in-depth interview (IDI)*: For children identified to have high BLL (e.g., >5 mcg/L), parent participants (anticipated at ~20) will be invited to have a home visit by the research team for observation of lead exposure risk factors within their home. These participants will also undergo an IDI to evaluate their understanding of lead reduction messages. Qualitative data insights obtained from IDIs will be used to refine messaging in Aim 2.

*Rationale for sample size.* Based on the prevalence of high BLL in ABC child participants at age 12 months (8.2%), a *sample of 350 in the pilot will be sufficient to identify ~20 children with high BLL.* Twenty participants in each phase of in-depth interviews will provide sufficient data to achieve data saturation.

## **Aim 1. Screening and Selection of Study Participants**

For Aim 1, participants will be drawn from the ABC cohort. A standardized questionnaire will be used to assess study eligibility.

## **Aim 1. Recruitment and Consenting Procedures**

*Recruitment procedures:* Study staff will be trained in proper techniques for approaching potential study participants. The ABC enrollment consent specifically describes re-contact for recruitment into future studies for which they may be eligible, and included a space for participants to opt in to giving permission for re-contact. Study staff will review the ABC consent documents and will attempt to approach only participants who have given written permission.

Study staff will contact these former ABC participants (e.g., by phone or will approach them in person at the Dandora II health facility), to let them know about the study. Interested parents will be scheduled for a visit at the study clinic at Dandora to learn more and undergo a brief structured eligibility screening questionnaire. ABC participants may also be recruited for LEIP at their ABC study visit, if they have not yet been exited from ABC.

*Consenting procedures*: For those who are eligible and wish to enroll in this study, written informed consent will be administered. Parents will be providing consent for participation on behalf of themselves and their child. Study staff will discuss the consent process using a standardized series of steps that conclude with an explanation of the consent form that will clearly state that subjects can refuse to participate at any time without consequences. Potential participants will be informed that participation is voluntary and participation or non-participation will in no way alter the nature of their health care services.

Study staff will provide an in-depth explanation of the purpose, procedures, risks and benefits of the study, in the language of the potential participant’s choosing. Informed consent discussions will take place in a quiet private setting with minimal distractions. The consent will be read aloud, and the potential participant will be given opportunity to ask questions. Parents will be encouraged to discuss participation in the study with their partner prior to enrollment.

A copy of the consent form describing in detail the study procedures and risks will be given to the participant.

If the participant is not literate, a witness unrelated to the study will be present. The participant will provide oral consent with a witnessed thumbprint or mark documented as consent.

## **Aim 1. Data Collection Procedures**

*Aim 1. Overview of visit schedule:* Participants may complete 1-5 study visits, including enrollment (Table 2). A subset will complete an in-depth interview (IDI) on the Lead Risk Survey and lead point of care (POC) test experience (e.g., 2 separate visits). In addition, for participants with high BLL, there will be a home visit, an IDI on the home visit, and a blood lead level re-check visit (e.g., 3 additional separate visits).

| Table 2. Overview of Aim 1 procedures and data collection. | | | | | |
| --- | --- | --- | --- | --- | --- |
| Visit | Enrollment (N=350) | Lead risk survey & POC test IDI (subset, N=20) | Home visit  (all with high BLL; N~20) | Home visit IDI (all with high BLL; N~20) | BLL Recheck (all with high BLL; N~20) |
| Procedures | All: Lead POC test  If high BLL: Venous blood draw | Interview | Home observation and messages  Collect environmental media, in situ XRF lead detection | Interview | Lead POC test  Venous blood draw |
| D*ata collection* | *All: Demo-graphics*  *Lead Risk Survey* | *Understandability, acceptability of the Lead Risk Survey and POC test* | *Lead Risk Survey* | *Understand-ability, acceptability of messages on lead exposure reduction* | *Knowledge, attitudes practices (e.g., Recall of messages, behavior change)* |
| In-depth interview, IDI; point of care, POC; blood lead level, BLL; X-ray fluorescence detector. | | | | | |

### *Aim 1 Enrollment Visit:*

1. Enrollment data collection. Following informed consent, parents will provide locator information and will be administered a questionnaire on demographics.
2. A child lead POC test will be performed. Two drops of blood (a fingerstick sample) will be needed for the test. If the BLL is high, a confirmatory test will be done, involving 2 additional drops of blood. The POC test will be done suing a LeadCare II device or similar.

Study staff will ensure careful washing of the child’s hand with soap and water and air drying, prior to cleaning with alcohol. Using sterile technique, a microlancet and capillary tube will be used to prick the child’s finger and draw approximately 50 uL of capillary blood. This will be loaded into the LeadCare device for a near-instant reading (3 min).

***Threshold for defining high blood lead level (BLL):*** We plan to use the WHO reference level of ≥ 5 ug/dL to define children with high BLL and eligible for follow up procedures unless stakeholders suggest an alternative lower reference level be used. For children with BLLs equal to or exceeding the reference level, **a repeat confirmatory fingerstick BLL** will be conducted. This is a practice used in the United States when timely confirmation using venous sample testing using sensitive laboratory techniques is not readily available. In these cases, two POC results ≥ the reference level are used to confirm the child’s BLL is high.

1. Venous blood draw for anemia and future lead testing using sensitive techniques. For any child who has a POC test confirmed high BLL, a venous blood sample will also be collected. This will be used to check for anemia, and archived for in-laboratory batch analysis using sensitive standardized laboratory techniques. Anemia testing is important because iron deficiency can increase absorption of lead, exacerbating toxicity. In-laboratory sensitive assays are important to allow measurement of the change in BLL from enrollment. The LeadCare II assay is not designed for precise quantitative analysis and does not allow quantitation of BLLs below 3.3 ug/dL. The venous sample will be collected using standardized sterile technique by a trained pediatric phlebotomist. To reduce discomfort, we will use a topical anesthetic for the venous blood sample collection. The sample collected will be approximately 0.5-3 mL (approximately one teaspoon) from each child.

Parents will receive their child’s anemia test result by phone or the next in-person visit, which-ever comes first.

1. Lead Risk Survey. All parent participants will be administered a brief survey on lead exposures (Lead Risk Survey), regardless of their child’s lead result.
2. Results and messaging on ways to reduce exposure to lead. Parent participants will be given standard post-test messaging on reducing lead exposure based on the responses from their survey. Parents of children with high BLL will be given additional tailored messaging. For children with a BLL below the reference level, the result will be provided to the parent, together with standard post-test messaging on reducing lead exposure. For parents whose child testing results in confirmed BLL equal to or above the reference level, targeted messaging on potential sources identified on the Lead Risk Survey will be provided.

***Referrals.*** For any child with BLL > 25 ug/dL) special referrals (e.g., determination and treatment of iron deficiency, early childhood development support) will also be provided. Protocols for these supports will be developed with guidance from the LEIP Study Advisory Group. The LEIP Advisory Group members represent national or international experts on pediatric environmental health/medicine & lead exposure assessment and mitigation, Kenyan pediatric clinical health care leadership and Kenyan health policy.

1. Lead Risk Survey, message and Lead POC Test In-Depth Interview. A random subset (N=20) of all participating parents will be recruited to participate in in-depth interviews in order to evaluate Lead Risk Survey and message understandability and acceptability and acceptability of the Lead POC Test. This will be scheduled according to the participant’s preference.

***In-depth interview procedures:*** Interviews will be conducted by study staff with specialized training in qualitative interview techniques and in the language of the participant’s choosing. Interviews will be audio-recorded for transcription and translation to English, as appropriate. Interviews will be conducted at a future time based on participant preference, and will be conducted in a location amenable to the interview subject (e.g., a private area within the study clinic).

1. Exit. Participants with **low BLL** will be thanked for their time and exited following after receiving their results and post-test messaging, unless they have been selected for a Lead Risk Survey and Lead POC Test In-depth Interview.

### *Aim 1 Follow-up Procedures:*

Follow-up procedures will only involve children with high BLL. Briefly, participating parents of these children will be offered a home visit that includes environmental sampling (described below).

1. Home visit. Home visits will be conducted within 2-4 weeks of the lead POC test, or according to the parent’s preference or the study team’s capacity. During the home visit, procedures will include:
2. The Lead Risk Survey will be re-administered and a Home Observational Checklist will be used to identify and discuss residential features and items in child’s home environment that may be influencing the high BLL.
3. Tailored messages on lead exposure risk reduction will be provided.

As possible, items and media that are suspected contributors (e.g., soil near the home where the child plays/spends time, house dust, cooking pots and utensils, cosmetics, spices, toys, painted walls and surfaces) will be tested for lead in situ using an XRF device and documented with photos. These devices can measure lead safely in a variety of household exposure media including dust and soil, painted walls, cookware, children’s toys and clothing, and cosmetics. GPS coordinates will be obtained.

d. Environmental media samples (e.g., soil, the child’s main water source) will be collected for analysis.

1. Home Visit In-depth Interview (IDI). Home visit participants will be asked to participate in IDIs on understandability and acceptability of risk reduction messages and participant anticipated facilitators and barriers to uptake of recommended exposure reduction measures. This will be conducted on the same day or an alternate time based on participant preference. Interviews will be conducted as described above under *“Lead Risk Survey IDI.”*
2. BLL re-check visits. Participants with high BLL will be offered a return to clinic for BLL re-checks at 6 months post lead POC test (a natural timepoint for ABC participants). The re-check will be administered using the POC testing protocol described above. A venous blood sample will be collected.
   1. Referrals as described above will be made for any child who is found to have a newly identified BLL > 25 ug/dL.
   2. Parents will respond to standardized questionnaires to assess their knowledge of exposure risk factors discussed at the time of BLL testing and the home visit and uptake of recommended reduction measures.
   3. Participants will be thanked for their time and exited following after receiving their results and post-test messaging, unless a referral for BLL >25 ug/dl is required.

Data collected as part of Aim 1 may inform Aim 2. Any meaningful changes to the protocols or data collection tools developed through Aim 1 will be provided to the UW HSD and the KNH – UoN ERC for review and approval as a modification, prior to beginning Aim 2 study procedures.

AIM 2

## **Aim 2. Overview**

Parent-child pairs will be offered child lead POC testing. Pairs with a child with a high BLL will be randomized to two groups. Both groups will receive an in-clinic lead exposure risk reduction messaging protocol. One group will also receive an at-home lead exposure risk reduction messaging protocol.

## **Aim 2. Study Area Description and Study Site**

Recruitment and enrollment will take place at the Dandora II Health Centre, and the Baba Dogo Health Centere. We may also conduct recruitment at other similar health centres in Nairobi, such as the Kariokor Health Centre, the Kariobangi Health Centre and the Eastlands Health Centre in Nairobi. These sites were selected to prioritize sites with a range of potential lead sources and/or where the study team has established study sites. Babo Ndogo Health Centre is located in the Industrial Area of Nairobi, serving clientele with diverse industrial occupational exposure. Our current ABC cohort study has a study office at Dandora II Health Centre.

## **Aim 2. Study Population**

Aim 2 will involve parent-child pairs coming for routine well-child-clinic services (such as growth monitoring, childhood vaccines) at the public health facilities described above.

### *Inclusion criteria*

- Child age is 12 to 72 months
- Parent or primary caregiver that is a biological relative is available to give informed consent for LEIP study
- Parent/caregiver is 18 years of age or older or is an emancipated minor
- Available for 9 months follow-up
- Willing to allow the collection of blood from the child for a lead POC test
- Willing to be randomized to one of two intervention groups

### *Exclusion criteria*

- Unwilling to be contacted for the purposes of tracing and follow-up

## **Aim 2 Sample Size and Power**

*Sample size:* 1,500 children will be enrolled and undergo POC BLL testing. We anticipate this sample size is required to achieve N = 100 with high BLL (e.g., ≥ 5 ug/dL), based on estimated of high BLL in ABC child participants at age 12 months (8.2%). Should we achieve our target of N = 100 randomized prior to enrolling 1,500 parent-child pairs, we will stop enrollment early.

Parents of these children with high BLL will be randomized to immediate messaging tailored per in-clinic survey alone vs the same protocol along with a home visit for home observational checklist + reinforced messaging per home findings (N = 50 in each arm). Follow up visits at 3 and 9 months will include surveys of message recall, behavior change to reduce exposure, and a BLL recheck.

*Power:* Primary outcomes will be recall of survey identified exposure risk factors and self-reported uptake of risk reduction behavior. Assuming 5% attrition and 10% attrition at the 3 and 9 month re-check visits, respectively, we anticipate n = 95 and n = 90 at re-check visits, respectively. Assuming n = 90, alpha = 0.05, a one-sided test, and an observed recall or uptake of ≥ 67% within an arm, we will have ≥ 80% power to exclude a recall or uptake of < 50%. For an observed recall or uptake of > 43% within an arm, we will have ≥ 80% power to exclude the possibility that recall / uptake is actually < 25%.

## **Aim 2. Screening and Selection of Study Participants**

For Aim 2, participants will be drawn from routine clientele accessing child vaccinations at each study site. A standardized questionnaire will be used to assess study eligibility.

## **Aim 2. Recruitment and Consenting Procedures**

*Recruitment procedures:* Parent-child pairs will be recruited at well-child vaccination clinics. Clinic staff will be trained in the study eligibility criteria and will be asked to help identify potentially eligible parent-child pairs and refer them to study staff. Clinic staff will be asked to read a brief recruitment script, and interested parents will be referred to study staff, who may provide more detailed information about the goals and procedures of the study. Interested parents will undergo a standardized structured questionnaire to assess study eligibility.

*Consenting procedures:* Consenting procedures will be conducted as described under Aim 1, given that the study population is similar, involving parent-child pairs identified at publicly accessible health facilities.

## **Aim 2. Data Collection Procedures**

*Aim 2. Overview of visit schedule:* Participants will receive a lead POC test at enrollment (Table 3). Participants whose children have low BLL will be exited following the lead POC test. Participants whose children have high BLL may complete up to 6 study visits, including enrollment. Participants with high BLL will have a randomization visit, a home visit (if randomized to home visit group), and two BLL re-check visits. A subset (N = 20) will participate in an in-depth-interview (IDI), described under Aim 3.

| Table 3. Overview of Aim 2 procedures and data collection. | | | | |
| --- | --- | --- | --- | --- |
| Visit | Enrollment (N=1,500) | Randomization  (if high BLL, N=100) | Home visit  (if randomized to home visit N=50) | BLL recheck at +3 and +9 months (all randomized N=100) |
| Procedures | Lead POC test | Venous blood draw  Randomize  (in-clinic messaging only vs in-clinic messaging + home visit and tailored messaging) | Home observation and messaging | Lead POC test  Venous blood draw |
| *Data collection* | *Demographics*  *Lead Risk Survey* |  | *Lead Risk Survey* | *Knowledge, attitudes, practices (eg. Recall of messages, behavior change)* |
| In-depth interview, IDI; point of care, POC; blood lead level, BLL | | | | |

### *Aim 2 Enrollment Visit:*

Enrollment data collection. Following informed consent, parents will provide locator information and will be administered a questionnaire on demographics.

1. A child lead POC test will be performed. Children will receive a lead POC test, as described under Aim 1. Briefly, two drops of blood (a fingerstick sample) will be needed for the test. If the BLL is high, a confirmatory test will be done, involving 2 additional drops of blood. The POC test will be done using a LeadCare II device or similar.
2. Lead Risk Survey. All parents will be offered a Lead Risk Survey and basic lead risk reduction messaging, regardless of their child’s BLL. We anticipate testing up to 1,500 children, in order to identify at least N=100 with confirmed BLL equal to or above the reference level for participation in a randomized trial.
3. Exit. Parents of children with **low BLL** will be thanked for their time and exited.
4. Randomization. Parents of children with **high BLL** will be offered randomization to in-clinic tailored risk reduction messaging alone vs the same protocol along with follow up home visit for home observation checklist survey with reinforced tailored messaging (N=50 in each arm). A randomization visit will be scheduled according to the parent’s preference. The home visit will be scheduled for 2 weeks within the initial randomization visit.

Blood draw. As in aim 1, a venous blood sample (0.5 to 3 ml) will be collected from trial participants for anemia testing, storage and later batched lead analysis using a standardized, highly sensitive laboratory-based method. If parents decline participation in the randomized trial, they may still be offered a blood draw so that their child can receive an anemia test.

Parents will receive their child’s anemia test result by phone or the next in-person visit, whichever comes first.

### *Aim 2 Follow-up Procedures (randomized participants only):*

1. Home visit. Participants randomized to the home visit will have a home visit within 2-4 weeks of the lead POC test or according to the parent’s preference or the study team’s capacity. During the home visit, procedures will include:

a. The Lead Risk Survey will be administered and a Home Observational Checklist will be used to identify and discuss residential features and items in child’s home environment that may be influencing the high BLL. Photos will be taken of household items, with the participant’s permission. No faces will be shown in the photos. GPS coordinates will be obtained.

1. Tailored messages on lead exposure risk reduction will be provided.
2. Follow-up BLL re-checks. All randomized participants will be scheduled for 3- and 9-month follow-up visits and lead POC tests. At these visits a venous blood sample will also be collected.
3. Knowledge, attitude and practice (KAP) survey. At the 3-month and 9-month BLL re-check visits, a standardized questionnaires will be administered to assess knowledge recall of risk factors identified at BLL testing, uptake of lead exposure risk reduction recommendations, and individual determinants related to these behaviors.
4. At the three month visit, a subset of participants (N = 20) will be invited to participate in an in-depth-interview on barriers, acceptability and understandability of lead exposure risk reduction in their children. (This is part of Aim 3 and is described in more detail below).
5. Exit. At the 9-month visit, participants will be thanked for their time and exited, following lead POC testing, venous blood collection and the KAP survey.

AIM 3

## **Aim 3. Overview**

Aim 3 will consist of qualitative data collection focusing on Aim 2 parent participants (in-depth interviews or IDIs) to understand the individual level barriers to uptake of behavior changes to reduce exposure to lead. Key informant interviews (KIIs) including stakeholders will be used to and structural level barriers to implementation of lead exposure interventions in order to inform policy.

## **Aim 3. Study Area Description and Study Site**

Recruitment and enrollment of parent participants will take place at LEIP follow-up study visits. These will be based at the Dandora II health centre, the Baba Ndogo health centre, Pumwani Hospital, and Mama Margaret Hospital.

## **Aim 3. Study Population**

Aim 3 will involve two groups:

1) A subset of parents whose children are identified to have high BLL in Aim 2.

*Inclusion criteria*

- Mothers or fathers of children with high BLL identified in Aim 2.
- Willing to provide informed consent for an in-depth interview.

*Exclusion criteria*

- Unwilling to be audio-recorded during the interview.

2) Key stakeholders who are involved in lead exposure reduction programs including LEIP providers (study staff), other frontline health workers mix of community health promotors, community health volunteers, nurses, clinical officers, pediatricians and supervisors), and other key persons (e.g., drawn from national and county level government entities, and institutions, including the Kenya Ministry of Health, Ministry of Environment, Council of Governors, the Nairobi City County, and key professional societies, e.g., the Kenya Paediatrics Association and the Kenya Chemical Society).

*Inclusion criteria*

- Either a community leader or community health worker, a policy maker in the health or environment sector, a clinician, or study staff involved in the LEIP study
- Age 18 years and above
- Willing to provide informed consent

*Exclusion criteria*

- Unwilling to be audio-recorded during the interview.

## **Aim 3. Sample Size and Power**

*Sample size:*

20 parents from Aim 2 will participate in in-depth interviews to understand barriers, acceptability and understandability of lead exposure risk reduction in their children.

20 key stakeholders will participate in key informant interviews to understand barriers and appropriateness of LEIP program components for scale up, future success.

*Power:* We anticipate these sample sizes will be sufficient to achieve data saturation.

## **Aim 3. Screening and Selection of Study Participants**

Parent participants will be drawn from the randomized trial of Aim 2. We will purposively include 10 participants from each arm and with varying child BLLs.

Stakeholders will purposively include a mix of health facility workers, supervisors and policymakers. A brief structured questionnaire will be administered to allow assessment of study eligibility.

## **Aim 3. Recruitment and Consenting Procedures**

*Recruitment procedures:* Parent participants in Aim 2 will be approached at a scheduled study visit and will be offered participation in in-depth interviews.

Health facility staff will be recruited from health facilities by study staff. In addition, relevant stakeholders including government officials and professional organization members will be identified by our team of co-investigators.

*Consenting procedures*:

Written informed consent will be administered. Study staff will explain to each potential participant the purpose, risks and benefits of the study. Informed consent discussions will take place in a quiet private setting with minimal distractions. Potential participants and will be given opportunity to ask questions and will be informed that their participation is voluntary and that participation or non-participation in the study will in no way alter the nature of health care services that they receive or their employment situation.

A copy of the consent form describing in detail the study procedures and risks will be given to the participant.

If the participant is not literate, a witness unrelated to the study will be present. The participant will provide oral consent with a witnessed thumbprint or mark documented as consent.

## **Aim 3. Data Collection Procedures**

Following informed consent, data collection will proceed immediately or will take place at a time of the participants choosing. For stakeholders, a brief enrollment questionnaire will be administered to gather socio-demographics and role in policy and health care.

The interviews will be conducted by study staff in a location amenable to the interview subject (a private area within the study clinic, other office, study office, public area, etc.). Interviews will be conducted by study staff with specialized training in qualitative interview techniques and in the language of the participant’s choosing and audio-recorded for transcription and translation to English, as appropriate.

In-depth interviews with parents (Aim 2 participants) will assess acceptability of messaging, facilitators, and barriers to individual uptake of messages related to lead exposure reduction, and determinants that may be related to these outcomes. Interview guide questions will explore knowledge acquisition, empowerment and self-efficacy, family dynamics and the socioecological context of these factors.

Key-informant interviews with stakeholders will assess barriers and facilitators to future implementation of LEIP from a policy and program planning perspective. Interview guides will explore design and packaging, complexity, strength and quality of the program, structural characteristics, networks and resources.

VARIABLES FOR AIMS 1, 2, and 3

Information and quantitative variables to be collected for Aims 1, 2 and 3 are listed below in Table 4.

| **Table 4. Summary of type and sample size for information and variables to be collected for Aims 1, 2 and 3** | | |
| --- | --- | --- |
| **Data type** | **Aim, Sample size** | **Variables** |
| **Pre-enrollment screening** | **Aim 1 Pilot: N=350** | Parent date of birth, child date of birth, availability for study follow-up duration, willing for child to provide blood |
|  | **Aim 2 Randomized Trial: N=1,500** | Same as above |
|  | **Aim 3: Stakeholders, N=20** | Age, role in organization/policymaking |
| **Identifiers** | **Aim 1 Pilot: N=350** | Participant names, contact information |
|  | **Aim 2 Randomized trial: N=1,500** | Same as above |
|  | **Aim 3: Stakeholders, N=20** | Same as above |
| **Enrollment question-naires** | **Aim 1 Pilot: N=350** | Socio-demographics, eg, marital status, education and indicators of socioeconomic status  Lead Risk Survey: Self-report of lead risk factors, e.g, household paint chipping/peeling, parent occupational exposure, cosmetics, cookware, drinking water sources, residential proximity to busy roadways, dumpsites, lead related industries, non-food products mouthed by the child, child feeding, child anthropometry from medical record |
|  | **Aim 2 Randomized trial: N=1,500** | Same as above |
| **Follow-up question-naires** | **Aim 1 Pilot: subset with child with high BLL, N~20** | Knowledge, attitudes, practices (KAP) related to lead exposure, e.g., likert questions on recall of risk factors identified at initial lead test, uptake of exposure reduction recommendations, feelings about the lead test, ability, plans, challenges, strategies in reducing lead exposure |
|  | **Aim 2 Randomized trial: subset with child with high BLL & randomized, N=100** | Same as above |
| **Point-of-care and laboratory tests** | **Aim 1 Pilot: N=350** | Enrollment lead point of care test result using fingerstick (capillary) sample |
|  | **Aim 2 Randomized trial: N=1,500** | Same as above |
|  | **Aim 1 Pilot: subset with child with high BLL, N~20** | Enrollment BLL and anemia using venous sample  Follow-up point of care test result using fingerstick (capillary) sample  Follow-up blood BLL and anemia using venous sample |
|  | **Aim 2 Randomized trial: subset with child with high BLL & randomized, N=100** | Same as above |
| **Home visit** | **Aim 1 Pilot: subset with child with high BLL, N~20** | Observed presence of chipping paint surfaces, glazed pottery cookware or dishware, aluminum pots, kohl; we will collect photos of household items. |
|  | **Aim 2 Randomized trial: subset with child with high BLL & randomized to home visit arm, N=50** | Same as above |
|  | **Aim 1 Pilot: subset with child with high BLL, N~20** | Lead concentration detected by XRF assessment of household surfaces, e.g., products, dust, and proximal soil, lead concentration in household samples, eg, water |
| **Parent in-depth interviews** | **Aim 1 Pilot: Randomly selected subset, N=20** | Understandability and acceptability of the Lead Risk Survey and messages. |
|  | **Aim 1 Pilot: subset with child with high BLL, N~20** | Understandability and acceptability of the risk reduction messages (Aim 1)  Understandability and acceptability of risk reduction messages and participant perspectives on facilitators and barriers to the uptake of recommended exposure reduction measures. |
|  | **Aim 3 Parents participating in randomized trial: N=20** | Open ended questions on on recall of risk factors identified at initial lead test, uptake of exposure reduction recommendations, feelings about the lead test, ability, plans, challenges, strategies in reducing lead exposure |
| **Key informant interviews** | **Aim 3 Stakeholders** | Open ended questions on barriers and facilitators to future implementation of LEIP from a policy and program planning perspective. Interview guides will explore design and packaging of messaging materials, complexity, strength and quality of the program, structural characteristics, networks and resources |

## **Referrals for Children with Concern for Need of Chelation**

The WHO and HICs recommend consideration of chelation therapy for children who have a BLL ≥ 45 ug/dL and ensure child can return to an environment without ongoing exposure. There are no equivalent measures in Kenya and this study is designed to inform future programs. We anticipate BLLs will not reach these very high ranges in our study population. If a child is determined to have a confirmed BLL ≥ 45 ug/dL, we will refer the child for a pediatric health care evaluation as well as inform the Poison Information and Management Centre at Kenyatta National Hospital.

## **Participant’s Results Sharing**

We will share blood lead results and anemia results with participants in real-time using results generated by POC blood lead testing protocols and hemoglobin tests. This information will be shared by the study team performing the test in person at the time of the clinical visit (lead) and by phone once test results are available (anemia).

At the time of sharing with parents their child’s blood lead level results, parents will be provided with a brief counseling session, guided by a script provided on a paper form that will also include their child’s blood lead level results. Using this tool and a pictoral job aid (e.g., a poster), we will provide counseling and health education on the clinical relevance of the results and how to reduce their child’s subsequent exposure.

## **Instruments, Materials, Supplies and Laboratory Procedures**

Portable hand-held direct reading instruments (e.g., X-ray fluorescence [XRF] detectors) can be used to investigate sources in and around a child’s home (e.g., soil, dust, paint, cookpots and other products). For the detailed home visit subgroup, suspected lead containing items and media (e.g., soil near the home where the child plays/spends time, house dust, cooking pots/utensils, cosmetics, spices, toys, painted surfaces) will be tested for lead in situ using a Bruker S1 Titan Handheld XRF analyzer (Bruker Scientific Instruments, Billerica, MA, USA), or equivalent. We will follow manufacturer instructions and standard protocols (e.g., Pure Earth 202327, modified as needed^[[38]](#endnote-38)^) and use standard reference materials for accuracy evaluation. A sample of the child’s main water source will be collected in a lead-free jar and stored for analysis.

The LeadCare II FDA-approved*^[[39]](#endnote-39)^* POC analyzers (result in 3 minutes) are a practical lead testing approach employed in studies in Africa and in HIC settings where access to laboratories is a barrier. Following established procedures *^31^* and using sterile technique, a micro lancet and capillary tube will be used to prick the child’s finger and draw an approximately 50 uL capillary sample which will be loaded into the LeadCare device for near-instant (3 min) result. Assays including standard references materials will be run as per protocol to ensure accuracy.

Venous blood will be subjected to microwave digestion, according to standard protocols in Dr. Were’s laboratory at the University of Nairobi and the digests will be used to measure lead using ICP-MS. Assays will be run in triplicate and in the same batch as standard reference materials and water blanks. Hemoglobin assays will also be done locally.

## **Training Procedures**

Study procedures: The study coordinator will train the study staff in study procedures, including the recruitment, enrollment/informed consent, and participant survey based data collection. Specific training modules will be used to train staff specifically in the protection of human subjects in research, maintaining confidentiality and privacy, and secure collection and storage of electronic data. All staff will complete a human subjects training course such as the Collaborative Institutional Training Initiative (CITI) program, an established program for human subjects training [https://about.citiprogram.org/].

Qualitative data collection and analysis: Co-Investigator Ferdinand Mukumbang will train the team on interview guide development, qualitative interviewing, transcript coding and analysis.

Point of care blood lead level testing:

mPI Catherine Karr, and co-investigators Faridah Were and Anne Riederer will lead the trainings on POC BLL testing using Lead Care devices. Standard operating procedures have been developed as part of Dr. Karr’s work in Washington State in the U.S.A and these will be adapted and used to guide the study staff on the proper technique in obtaining a capillary sample to avoid lead contamination and using sterile techniques. Moreover, the team will be trained to provide confirmatory testing, where appropriate, document the test results, and to communicate the results to parents in a sensitive manner.

## **Quality Assurance Procedures**

For LeadCare, quality assurance procedures include following manufacturer instructions for reagent controls and storage and instrument calibration and maintenance. Certified reference materials will be run each day. Collected venous blood samples will be archived for confirmatory analysis using ICP-MS or equivalent.

Data will be collected using the electronic database REDCap, which allows immediate access and review of data collection. Interval summaries on a weekly or monthly schedule will enable us to review enrollment progress.

In-depth interview/key informant interview (IDI/KII) audio files and interviewer field notes will be uploaded to secure cloud-based storage such as UW OneDrive immediately after the participant IDI/KII concludes. These files will be transcribed and translated within a week of the IDI/KII. The process of audio file and field note upload, transcription, and translation will be monitored by the study coordinator.

The study coordinator will be available at all times to address staff concerns. At regular team meetings, we will review enrollment progress, data completeness and validity, themes arising from concurrent analysis, and address any concerns from the study sites.

ETHICAL CONSIDERATIONS

## **Ethical Standard**

The investigators will ensure that this study is conducted in full conformity with the principles set forth in the Declaration of Helsinki and The Belmont Report: Ethical Principles and Guidelines for the Protection of Human Subjects of Research, as drafted by the US National Commission for the Protection of Human Subjects of Biomedical and Behavioral Research (April 18, 1979) and codified in 45 CFR Part 46 and/or the ICH E6.

## **Institutional Review Board**

The protocol, informed consent forms, recruitment materials and all data collection tools will be submitted to the KNH ERC and UW IRB for review and approval separately at each. Approval of both the protocol and the consent forms must be obtained from both the KNH ERC and UW IRB before any participant is enrolled. Any amendment to the protocol will require review and approval by the ERC and IRB before the changes are implemented in the study.

## **Potential Risks and Benefits**

Confidentiality: There is a non-negligible risk of loss of confidentiality of sensitive medical information. All personnel will have been trained in the Protection of Human Subjects before they begin working on the study. Our field team has more than 15 years of experience managing women and children in the setting of clinical trials and will take every precaution to protect participants’ and their parents’ confidentiality. For home visits, study staff will wear plain clothes, will not discuss participation in a research study with anyone other than the study participant, and will offer home visits at a time that is of the participant’s choosing.

Privacy: There is a need to maintain study participant privacy. Study staff will make all efforts to ensure the privacy of participants. For interviews with parents, interviews will be done in a private health care setting. For health care workers/stakeholders, key informant interviews will occur in a neutral private location of the participants preference, which could include quiet private area in the recruitment clinic or office, at the participant’s home, on the phone, or via teleconference on a platform like zoom. At clinic or office visits, a private room will be used for the key informant interview. If phone call or teleconference are used, the study team will ensure that both the interviewer and study participant are in quiet private areas prior to commencing.

Sensitive questions: Standardized questionnaires for parents will include questions about maternal and infant health history, which may include sensitive questions. The study team will make every effort to ensure confidentiality and privacy of study participants.

Phlebotomy: Phlebotomy can cause pain and bruising. We will use a topical anesthetic to reduce the pain associated with blood draw for the children. The topical anesthetic may cause temporary redness or irritation of the skin in the area where it was applied.

Lead testing: Learning about a potential or actual pediatric lead exposure may cause parents stress. Should we identify any children who have blood lead levels above 25 ug/dL, we will provide a referral to the KNH tertiary referral hospital for specific management. Additional considerations include timeliness of providing child blood lead level information to parents, quality assurance and quality control in blood lead testing procedures. We will ensure that study staff are trained in each of these elements important for pediatric blood lead level testing.

Coercion: Despite every attempt by staff to ensure non-coercion during the informed consent and enrollment process, there is a non-negligible risk of participants feeling pressure to enroll in research studies.

Time Discomfort: Participants may experience some slight discomfort or stress because of the time taken to conduct the in-depth interviews/key informant interviews or blood lead level testing. However, these discomforts are anticipated to be minor.

Justification for randomization scheme: There is a need to identify approaches for lead exposure risk reduction in LMICs that maximize potential for **adoption and long-term sustainability**. Although a package including a home visit may be most effective, an in-clinic risk survey may be the most feasible and warrants evaluation. Our randomized design allows us to evaluate both models in similar groups of parent-child pairs, with the goal of informing policymakers on the minimum implementable package appropriate for this context.

POTENTIAL BENEFITS

**Direct benefits**

Parents will have access to child blood lead level testing, which is currently generally unavailable to most children in Kenya. Parents will also have access to interviews, messaging and home visits conducted by study staff who are trained in providing guidance on ways to avoid pediatric lead exposure.

Health facility staff will have access to training on blood lead level testing and will have practical opportunity to practice using point of care blood lead level test devices.

**Indirect benefits**

Since access to BLL testing for young children is limited in Kenya, this study with have a benefit on the societal level, given that this study may contribute to public awareness on lead as a neurotoxic substance and ways to avoid lead. The knowledge generated could have a beneficial impact on reducing lead exposures in parents and young children and may have future impact on policy and structural level changes to reduce lead exposures.

## **Informed Consent Process**

Informed consent is a process that is initiated prior to the individual agreeing to participate in the study and continues throughout study participation. Consent forms will be reviewed and approved by the ERC and IRB before use. Consent forms will be available as English and Kiswahili versions. We will only enroll participants who are able to communicate in English or Kiswahili. If the individual is illiterate, study staff will read the consent form out loud to the individual, and another staff member will be asked to witness their answers to questions regarding the consent process and their signing of the form. Parents and clinicians will provide consent for participation on behalf of themselves.

Study staff will explain the research study to the participant and answer any questions that may arise. A consent form describing in detail the study procedures and risks will be given to the participant. Extensive discussion of risks and possible benefits of study participation will be provided to participants. The participant will read (or have the form read to them) and the staff member administering consent will ask questions of the participant to assess comprehension of the study. The participant will sign the informed consent document prior to any study-related assessments or procedures.

Participants will be given the opportunity to discuss the study with their partners or think about it prior to agreeing to participate. They may withdraw consent at any time throughout the course of the study. All participants can refuse any research procedures at any time. If there is any reluctance to undergo study procedures, we will revisit consent and remind them that all participation and procedures are voluntary and ask if they wish to continue participation. If participants do not present for the in depth interview or follow up visits after 3 successful tracing contacts are made, we will consider this withdrawal from the study.

A copy of the signed informed consent document will be given to participants. The rights and welfare of parent participants will be protected by emphasizing to them that the quality of their clinical care will not be adversely affected if they decline to participate in this study. The rights and welfare of clinician participants will be protected by emphasizing to them that their clinical practice will not be adversely affected if they decline to participate in this study. The consent process will be documented in the research record.

PARTICIPANT CONFIDENTIALITY AND PRIVACY

**Confidentiality**

To protect participant confidentiality, all key personnel will be trained in the Protection of Human Subjects before they begin working on the study. Study staff will take every precaution to protect participants’ confidentiality.

*Participant identifiers:* Name, address, phone number, birthdate, whether the participant is a parent or key informant, and child age (if a parent) will be attached to a coded study ID number. The key to this link will be stored in paper format and secured with lock and key. Only study staff will have access to this link-log. After data collection and initial data analysis are complete, we will retain the link between study code numbers and direct identifiers for the length of time required by US state and/or federal law, and Kenyan law, to maintain integrity of study data. After this period, the links to identifiable data for this cohort will be destroyed.

*All Study data* will be collected and stored on secure password-protected UW cloud-based storage, such as on UW OneDrive, on an electronic REDCap database on study tablets or laptops, or in paper under lock and key. All data will be password protected or secured with lock and key. Data will only be retrievable by study staff.

**Privacy**

The in-depth interviews/key informant interviews will occur in a neutral private location of the participants preference, which could include quiet private area in the recruitment clinic or office, at the participant’s home, on the phone, or via teleconference on a platform like zoom. Study staff will make all efforts to ensure the privacy of participants. At home visits, this will include wearing plain clothes. At clinic or office visits, a private room will be used for the interviews. If phone call or teleconference are used, the study team will ensure that both the interviewer and study participant are in quiet private areas prior to commencing.

## **Future Use of Stored Specimens and Other Identifiable Data**

Environmental media (e.g., water) samples may be used for analysis of additional contaminants in the future.

Child blood samples may be used for analysis of additional analytes (e.g., environmental contaminants, nutrients) in the future.

## **Retention Plan**

Parent participant retention procedures only apply to a subgroup of Aim 1 and a subgroup of Aim 2 participants.

Aim 1 participants are involved in the ABC study. The ABC study employs various strategies to enhance retention in the parent study, and similar approaches will be used for LEIP. Enrollees will be asked to complete an updated contact form during the enrollment visit to enable follow-up. Additionally, with participants’ permission, study staff may accompany the participant to their residence after the enrollment visit to record residence information for contact tracing. Participants are provided reminder cards and SMS messages for study clinic appointments and ABC study staff conducts active tracing for missed visits through phone calls and home visits.

In this proposal, Aim 1 participants who are screened with a blood lead test may go on to participate in an in-depth interview or a follow up home visit sub study followed by an in-depth interview. We will aim to reduce participant burden for these participants by scheduling the interview and home visit at the participant’s convenience. Incentives of 800 Kenyan shillings (~$8 USD) will be provided as reimbursement for participant time.

Aim 2 involves participant retention for children enrolled in a two arm intervention. Intervention participants will be asked to participate in two follow up visits at the clinic.

The study team has a long history of high retention of parent participants and their young children in cohorts in Nairobi. At the point of informed consent, the parents will be explained the importance of attending follow-up BLL recheck visits, and will schedule the follow-up visits at the time of testing. For these follow up blood lead test visits that occur in the clinic, all participants will receive 800 Kenyan shillings (approximately 8 US dollars) as reimbursement for travel and effort for each clinic visit. This may enhance retention. This will include the IDIs for parents for Aim 3.

Detailed contact information will be collected for each participant, including primary and secondary phone numbers, and household address (or information about location of the household). This contact information will be kept confidential and stored securely. Reminders of upcoming visits will be via phone calls and/or SMS. If a parent fails to return for scheduled follow-up visits, the study nurse will contact the participant via cell phone. If the study nurse is not able to contact the participant via cell phone, or if no telephone number is provided, study counselors will trace the participant at their household within 2 weeks of the scheduled follow-up time.

DATA MANAGEMENT

All data management activities will be co-overseen by the Seattle-based and Kenya-based data management team. Our teams have collective over 20 years of experience.

## **Types of Data**

Study participant self report data will be collected using an electronic REDCap database on study tablets or laptops [<https://www.iths.org/investigators/services/bmi/redcap/>]. Enrollment data may also be abstracted from medical records.

Audio files and field notes: In-depth and key informant interviews (IDI/KII) will be recorded on a portable audio recorder. Study staff will take field notes during or immediately after each IDI/KII. Audio files and field notes will be uploaded to a secure cloud-based password-protected server such as UW OneDrive. Once the audio files have been uploaded to the secure server, they will be deleted from the audio recorder.

Transcripts: Study staff will transcribe IDI/KII audio files and, if applicable, translate them to English. These transcripts will be stored on a secure cloud-based password-protected server such as UW OneDrive.

## **Data Entry and Cleaning**

Data will be entered into a electronic REDCap database on study tablets or laptops [<https://www.iths.org/investigators/services/bmi/redcap/>]. REDCap is HIPAA-compliant for the storage and transfer of protected health information, and uses SSL encrypted data transfer that is equivalent to the level of security used by banks. The database will be password protected. Data will only be retrievable by study staff.

## **Data Storage**

Data will be stored in the study password-protected REDCap database. Audio files, transcripts, and field notes will be stored on secure password-protected UW cloud-based storage, such as on UW OneDrive. A dedicated data team, consisting of a Nairobi data team and a Seattle-based data management and analysis team, will be responsible for the entry, management, and monitoring of study data. The Nairobi team will communicate frequently with the Seattle-based team for reporting, study monitoring, and analysis.

## **Study Records Retention**

After data collection and initial data analysis are complete, we will retain the link between study code numbers and direct identifiers for the length of time required by US state and/or federal law, and Kenyan law, to maintain integrity of study data. After this period, the links to identifiable data for this cohort will be destroyed.

ANALYSIS PLAN

**Aim 1 primary outcomes will be survey understandability and acceptability, agreement between in-clinic and at-home administered survey items and understandability and acceptability of risk reduction messages**. Qualitative analysis of understandability and acceptability. Transcripts of the interviews will be fed into the Dedoose data management tool for analysis. We will conduct a rapid analysis to summarize themes on understandability and acceptability of survey items and messages. The rapid analysis will also explore facilitators and barriers to uptake of recommended exposure reduction measures, and individual level determinants associated with uptake (e.g., empowerment, self-efficacy). Using exit interview transcripts, we will use a hybrid inductive and deductive thematic analysis approach based on our *a priori* selected CFIR constructs. Two coders will independently code transcripts, and generate a comprehensive codebook based on the CFIR constructs and conduct a thematic analysis. If new constructs emerge during our predominantly deductive coding, we will include them inductively. Agreement between in-clinic and at home surveys. Weekly field and co-investigator team meeting discussions will review any observed non-agreement between data from in-clinic and at-home surveys for refinement of this survey in Aim 2.

**Secondarily,** we will estimate the proportion of mothers (self-report on in-clinic surveys) and assessed homes with identified actionable targets, e.g., suspect household products, including cosmetics, chipped paint, potential occupational exposure, or other lead containing items observed using direct read instrumentation). We will describe the number and types of actionable targets identified for each participant. These data will enhance our adapted survey and messaging protocols for Aim 2. We will summarize descriptive statistics for standardized questionnaires on recall of identified risk factors, behavior change (e.g., proportions with recall of all or some factors vs. proportions with recall of some or no factors) and Risk, Ability and Attitude (e.g,, proportions with strong agreement vs. weak or no agreement). We will select questions with higher variability, and we will refine our questionnaires as appropriate for use in Aim 2.

**Aim 2 primary outcomes will be recall of risk reduction messages, and self-report risk reduction behavior. Exploratory outcomes will be uptake of the BLL re-check and change in BLL.** Balance between arms. We will compare baseline characteristics between randomization arms to evaluate balance of demographic and risk survey characteristics. We will use chi-square tests for dichotomous variables and rank sum tests or t-tests, as appropriate, for continuous variables.

**Recall of risk reduction messages and change in risk reduction behavior.** We will describe the proportion of parents with recall of all or most of their exposure factors identified on their surveys, within each arm and by child BLL. Similarly, we will describe proportions of parents self-reporting uptake of at least one of their risk reduction recommendations within arms and by BLL. We hypothesize that recall and behavior change will be moderate (e.g., greater than 50% in both arms), and that recall and behavior change will be highest for parents in the high BLL strata. Secondarily, we will explore potential differences in outcomes between arms, acknowledging that we will have limited ability to exclude small differences. Finally, we will describe the types of messaging recommendations taken up and not taken up by participants.

**Analysis of implementation strategy mechanisms.** We will summarize and explore differences in scores (e.g., for items using likert scales) for hypothesized individual determinants that may help explain uptake of each implementation strategy mechanism (e.g., knowledge acquisition, empowerment and self-efficacy, as well as male partner involvement and parent sex). Specific indicators will include perceived vulnerability, perceived risk, attitude regarding cost of lead exposure reduction, and perceived ability to take up messages). We hypothesize that parents with uptake of risk reduction behavior will have higher perceived vulnerability and risk, and greater perceived ability to take up messages compared with parents with lower uptake.

We anticipate that scores for individual determinants assessed using our questionnaires will be non-normally distributed and we will explore the relation between these cofactors (e.g., Risk, Attitude and Ability) and uptake of exposure reduction measures using non-parametric tests. We will use Wilcoxon rank-sum tests and log binomial regression or modified Poisson regression with robust errors, as appropriate to explore the relation between Risk, Attitude and Ability scores and uptake of risk reduction messages.

**Exploratory outcomes will be uptake of BLL recheck and change in BLL at 3 and 9 months.**

Return for a re-check. Return for a re-check visit is an indicator of parent awareness of importance of reducing lead exposure. We will summarize the proportions of parents returning for each BLL re-check visit for each arm, and compare to our benchmark of 50%, as for primary outcomes. We hypothesize that re-check will be high for both groups, and that re-check will be higher in parents with higher child BLLs. We acknowledge this outcome may have less variability across arms and may be subject to bias, given that participants will be provided a small reimbursement at each clinic visit.

We consider BLL change to be a distal indicator of effectiveness. In Lumumba et al,*^41^* standard deviations (SDs) were 15.5 ug/dL for maternal and 1.9 ug/dL for cord BLL. We anticipate that the SDs for child BLL will be lower than for adult women but higher than for cord samples.

**Aim 3 will evaluate individual and structural barriers to risk reduction and strategies for future lead prevention.**

**Individual and structural barriers.** After the 3-month re-check visit, we will administer Parent In-Depth Interviews in a subset of parents to further probe acceptability of messaging, facilitators, and barriers to individual uptake of messages related to lead exposure reduction, and determinants that may be related to these outcomes. We will aim to include parents with varying entry child BLLs and self-reported risk reduction behavior. We will use both select CFIR constructs and related interview questions and RANAS interview questions as guides. Interview guides will explore knowledge acquisition, empowerment and self-efficacy, family dynamics and the socioecological context of these factors, to enable mapping of these factors according to structural, community, household, individual and habitual levels.*^[[40]](#endnote-40)^* Interviews will be conducted in participant’s preferred language. Data analysis will be guided by a hybrid deductive-inductive thematic content analysis informed by the CFIR framework.

STUDY RESULTS DISSEMINATION PLAN

Closeout Meetings & Stakeholder Meeting: At the end of the study, results will be shared with the study sites (staff, administration) via a written report and/or oral presentation. We will share the results with representatives from Kenya Ministry of Health, the Nairobi City County, other government offices, and relevant professional organizations at a final stakeholder meeting.

Manuscripts & Conference Presentation: All findings will be analyzed, complied into manuscripts and submitted for peer-reviewed publication, with authorship agreed upon by the study investigators. We will additionally aim to present data to at least one international conference.

ITEMIZED BUDGET

| **Category** | **5-Year Total Direct Costs ($USD)** |
| --- | --- |
| Personnel Costs (Subtotal) | $834,692 |
| Consultant Services (including benefits, ASR, NSSF, UWK costs, ERC fees) | $17,000 |
| Travel for Investigators | $50,819 |
| Supplies and Materials | $50,210 |
| Equipment | $21,250 |
| Subawards/Consortium/Contractual Costs | $1,170,474 |

STUDY TIMELINE

|  | **Year 1** | | **Year 2** | | **Year 3** | | **Year 4** | | **Year 5** | |
| --- | --- | --- | --- | --- | --- | --- | --- | --- | --- | --- |
| Protocol and tools development |  |  |  |  |  |  |  |  |  |  |
| Obtain human subjects approval |  |  |  |  |  |  |  |  |  |  |
| Enroll and conduct follow-up for Aim 1 |  |  |  |  |  |  |  |  |  |  |
| Enroll, randomize and conduct follow-up for Aim 2 |  |  |  |  |  |  |  |  |  |  |
| Parent and stakeholder interviews for Aim 3 |  |  |  |  |  |  |  |  |  |  |
| Analysis and manuscripts |  |  |  |  |  |  |  |  |  |  |

**Study Closure Plan & Procedure**

At study closure, results will be communicated to each clinical site and to the Kenya Ministry of Health and the Nairobi City County via a written report and/or oral presentation. We will be available to answer questions from staff, administration, and leadership.

After their blood lead testing, most participants will not require follow-up for potential lead exposure and will not be longitudinally followed. For participants suspected to have lead exposure, the team will provide referrals or follow-up care.

STUDY LIMITATIONS

**Limitations and how to minimize limitations**

**Modifiable lead exposure sources in a resource constrained context.** Modifiable factors discerned may indicate avoiding certain cosmetics, replacing lead-containing cookpots, avoiding play in contaminated soil, or covering lead-containing paint with lead-free paint. Some of these may be beyond the means of families, others achievable. To ensure sustainable resources, the research team is committed to ongoing stakeholder discussions to inform future program needs; participant data in Aims 1 and 3 will provide perspectives on barriers and how to overcome them. **Participation and retention in LEIP.** Lead is an invisible health threat not readily attributed to lead in affected individuals (e.g., impaired cognition, learning difficulties, behavioral problems). We provide pre-test messaging to enhance interest through knowledge attainment, and in Aim 2 lead testing is offered as part of routine health care, fostering buy-in. In addition, our modality provides immediate BLL results -- more motivating than delayed result approaches. **Factors affecting will for** **change.** Macroeconomic pressures driving food insecurity and limited government resources may dilute political will for policy level change, despite clear evidence that some children are exposed to dangerous levels of lead. However, Kenya’s recent success in national regulations phasing out lead in paint*^11^* demonstrates momentum.

REFERENCES

1. . Kenya National Bureau of Statistics. 2019 Kenya Population and Housing Census, Volume I: Population by County and Sub-County. Nairobi:Kenya National Bureau of Statistics, November 2019 [Available: knbs.or.ke/?wpdmpro=2019-kenya-population-and-housing-census-volume-i-population-by-county-and-sub-county; accessed 4 March 2023]. [↑](#endnote-ref-1)
2. . Ernst KC, Phillips BS, Duncan BD. Slums are not places for children to live: vulnerabilities, health outcomes, and possible interventions. Adv Pediatr. 2013;60(1):53-87. doi: 10.1016/j.yapd.2013.04.005. Epub 2013 Jul 17. PMID: 24007840; PMCID: PMC7112084. [↑](#endnote-ref-2)
3. . Lilford RJ, Oyebode O, Satterthwaite D, Melendez-Torres GJ, Chen YF, Mberu B, Watson SI, Sartori J, Ndugwa R, Caiaffa W, Haregu T, Capon A, Saith R, Ezeh A. Improving the health and welfare of people who live in slums. Lancet. 2017 Feb 4;389(10068):559-570. doi: 10.1016/S0140-6736(16)31848-7. Epub 2016 Oct 16. PMID: 27760702. [↑](#endnote-ref-3)
4. . Kimani SK. Exposure to Pollutants and Health of Women Waste Pickers at Dandora Dumpsite in Nairobi, Kenya. Int J Innov Res Dev. 2021;10(6). doi:10.24940/IJIRD/2021/V10/I6/JUN21049. [↑](#endnote-ref-4)
5. . WHO guideline for the clinical management of exposure to lead. Geneva: World Health Organization; 2021. Licence: CC BY-NC-SA 3.0 IGO. [↑](#endnote-ref-5)
6. . UNICEF/Pure Earth. Toxic Truth: Children’s exposure to lead pollution undermines a generation of future potential. 2nd Edition. unicef.org/media/109361/file/The%20toxic%20truth.pdf; accessed 24 February 2024. [↑](#endnote-ref-6)
7. . Attina TM, Trasande L. Economic costs of childhood lead exposure in low- and middle-income countries. Environ Health Perspect. 2013 Sep;121(9):1097-102. doi: 10.1289/ehp.1206424. Epub 2013 Jun 25. PMID: 23797342; PMCID: PMC3764081. [↑](#endnote-ref-7)
8. . Zajac L, Kobrosly RW, Ericson B, Caravanos J, Landrigan PJ, Riederer AM. Probabilistic estimates of prenatal lead exposure at 195 toxic hotspots in low- and middle-income countries. Environ Res. 2020 Apr;183:109251. doi: 10.1016/j.envres.2020.109251. Epub 2020 Feb 15. PMID: 32311907; PMCID: PMC7176741. [↑](#endnote-ref-8)
9. . Ericson B, Hu H, Nash E, Ferraro G, Sinitsky J, Taylor MP. Blood lead levels in low-income and middle-income countries: a systematic review. Lancet Planet Health. 2021 Mar;5(3):e145-e153. doi: 10.1016/S2542-5196(20)30278-3. Erratum in: Lancet Planet Health. 2021 Nov;5(11):e765. PMID: 33713615. [↑](#endnote-ref-9)
10. . National Toxicology Program. NTP monograph on health effects of low-level lead. NTP Monogr. 2012 Jun;(1):xiii, xv-148. PMID: 23964424. [↑](#endnote-ref-10)
11. . Ondayo MA, Simiyu GM, Raburu PO, Were FH. Child Exposure to Lead in the Vicinities of Informal Used Lead-Acid Battery Recycling Operations in Nairobi Slums, Kenya. J Health Pollut. 2016 Dec 22;6(12):15-25. doi: 10.5696/2156-9614-6.12.15. PMID: 30524801; PMCID: PMC6221501. [↑](#endnote-ref-11)
12. . Were FH, Moturi MC, Gottesfeld P, Wafula GA, Kamau GN, Shiundu PM. Lead exposure and blood pressure among workers in diverse industrial plants in Kenya. J Occup Environ Hyg. 2014;11(11):706-15. doi: 10.1080/15459624.2014.908258. PMID: 24690073. [↑](#endnote-ref-12)
13. . Were FH, Kamau GN, Shiundu PM, Wafula GA, Moturi CM. Air and blood lead levels in lead acid battery recycling and manufacturing plants in Kenya. J Occup Environ Hyg. 2012;9(5):340-4. doi: 10.1080/15459624.2012.673458. PMID: 22512792. [↑](#endnote-ref-13)
14. . Otieno J, Kowal P, Mąkinia J. Monitoring Lead Concentration in the Surrounding Environmental Components of a Lead Battery Company: Plants, Air and Effluents-Case Study, Kenya. Int J Environ Res Public Health. 2022 Apr 25;19(9):5195. doi: 10.3390/ijerph19095195. PMID: 35564589; PMCID: PMC9103765. [↑](#endnote-ref-14)
15. . Maina EG, Gachanja AN, Gatari MJ, Price H. Demonstrating PM2.5 and road-side dust pollution by heavy metals along Thika superhighway in Kenya, sub-Saharan Africa. Environ Monit Assess. 2018 Mar 27;190(4):251. Doi: 10.1007/s10661-018-6629-z. PMID: 29582158. [↑](#endnote-ref-15)
16. . Njoroge GK, Njagi EN, Orinda GO, Sekadde-Kigondu CB, Kayima JK. Environmental and occupational exposure to lead. East Afr Med J. 2008 Jun;85(6):284-91. doi: 10.4314/eamj.v85i6.9626. PMID: 18817025. [↑](#endnote-ref-16)
17. . Ngo NS, Gatari M, Yan B, Chillrud SN, Bouhamam K, Kinneym PL. Occupational exposure to roadway emissions and inside informal settlements in sub-Saharan Africa: A pilot study in Nairobi, Kenya. Atmos Environ (1994) 2015; 111:179-184. [↑](#endnote-ref-17)
18. . Ngueta G, Ndjaboue R. Blood lead concentrations in sub-Saharan African children below 6 years: systematic review. *Trop Med Int Health* 2013; 18(10):1283-1291. [↑](#endnote-ref-18)
19. . Kinuthia GK, Ngure V, Beti D, Lugalia R, Wangila A, Kamau L. Levels of heavy metals in wastewater and soil samples from open drainage channels in Nairobi, Kenya: community health implication. Sci Rep. 2020 May 21;10(1):8434. doi: 10.1038/s41598-020-65359-5. Erratum in: Sci Rep. 2020 Jul 7;10(1):11439. PMID: 32439896; PMCID: PMC7242368. [↑](#endnote-ref-19)
20. . Njuguna SM, Yan X, Gituru RW, Wang Q, Wang J. Assessment of macrophyte, heavy metal, and nutrient concentrations in the water of the Nairobi River, Kenya. Environ Monit Assess. 2017 Aug 16;189(9):454. doi: 10.1007/s10661-017-6159-0. PMID: 28815343; PMCID: PMC5559568. [↑](#endnote-ref-20)
21. . Olewe TM, Mwanthi MA, Wang'ombe JK, Griffiths JK. Blood lead levels and potential environmental exposures among children under five years in Kibera slums, Nairobi. East Afr J Public Health. 2009 Apr;6(1):6-10. doi: 10.4314/eajph.v6i1.45734. PMID: 20000055. [↑](#endnote-ref-21)
22. . Riederer AM, Adrian S, Kuehr R. Assessing the health effects of informal e-waste processing. J Health Pollut. 2013; 3(4):1-3. //doi.org/10.5696/2156-9614-3.4.1. [↑](#endnote-ref-22)
23. . Clune AL, Falk H, Riederer AM. Mapping Global Environmental Lead Poisoning in Children. J Health Pollut 2011;1(2):14-23. [↑](#endnote-ref-23)
24. . Nakata H, Nakayama SM, Ikenaka Y, Mizukawa H, Ishii C, Yohannes YB, Konnai S, Darwish WS, Ishizuka M. Metal extent in blood of livestock from Dandora dumping site, Kenya: Source identification of Pb exposure by stable isotope analysis. Environ Pollut. 2015 Oct;205:8-15. doi: 10.1016/j.envpol.2015.05.003. Epub 2015 May 19. PMID: 25997160. [↑](#endnote-ref-24)
25. . Mutune A, Makobe, MA, Abukutsa-Onyango, MOO. Heavy metal content of selected African leafy vegetables planted in urban and peri-urban Nairobi, Kenya. Afr J Environ Sci Technol 2014; (1):66e74. [↑](#endnote-ref-25)
26. . Dickinson NM, Lepp NW, Surtan GT. Lead and potential health risks from subsistence food crops in urban Kenya. Environ Geochem Health. 1987 Jun;9(2):37-42. doi: 10.1007/BF01686173. PMID: 24214184. [↑](#endnote-ref-26)
27. . Pure Earth. 2023. Lead in Consumer Goods: A 25 Country Analysis of Lead (Pb) Levels in 5,000+ Products and Foods. pureearth.org/rapid-market-screening-program/; accessed 24 February 2024. [↑](#endnote-ref-27)
28. . Etiang' NA, Arvelo W, Galgalo T, Amwayi S, Gura Z, Kioko J, Omondi G, Patta S, Lowther SA, Brown MJ. Environmental Assessment and Blood Lead Levels of Children in Owino Uhuru and Bangladesh Settlements in Kenya. J Health Pollut. 2018 Jun 11;8(18):180605. doi: 10.5696/2156-9614-8.18.180605. PMID: 30524854; PMCID: PMC6239056. [↑](#endnote-ref-28)
29. . CDC [U.S. Centers for Disease Control and Prevention]. 2022. Blood Lead 2011 – 2018: Geometric mean and selected percentiles of blood concentrations (in µg/dL) for the U.S. population from the National Health and Nutrition Examination Survey. cdc.gov/exposurereport/data_tables.html; accessed 24 February 2024. [↑](#endnote-ref-29)
30. . FDA [U.S. Food and Drug Administration]. Update - February 15, 2023: Magellan Diagnostics resumed distribution of its LeadCare II test kits in February 2022, and resumed distribution of its LeadCare Plus and LeadCare Ultra test kits in October 2022. [Available: fda.gov/medical-devices/medical-device-recalls/magellan-diagnostics-recalls-leadcare-ii-leadcare-plus-and-leadcare-ultra-blood-lead-tests-due-risk; accessed 4 March 2023]. [↑](#endnote-ref-30)
31. . Nakata H, Nakayama SMM, Yabe J, Muzandu K, Toyomaki H, Yohannes YB, Kataba A, Zyambo G, Ikenaka Y, Choongo K, Ishizuka M. Assessment of LeadCare® II analysis for testing of a wide range of blood lead levels in comparison with ICP-MS analysis. Chemosphere. 2021 May;271:129832. doi: 10.1016/j.chemosphere.2021.129832. Epub 2021 Feb 2. PMID: 33736222. [↑](#endnote-ref-31)
32. . EPA [U.S. Environmental Protection Agency]. SW-846 Test Method 6200: Field Portable X-Ray Fluorescence Spectrometry for the Determination of Elemental Concentrations in Soil and Sediment. [Available: epa.gov/sites/default/files/2015-12/documents/6200.pdf; accessed 4 March 2022]. [↑](#endnote-ref-32)
33. . Washington State Department of Health. A Targeted Approach to Blood Lead Screening in Children, Washington State, 2015 Expert Panel Recommendations. DOH 334-383, May 2016 [Available: doh.wa.gov/sites/default/files/legacy/Documents/Pubs//334-383.pdf; accessed 4 March 2023]. [↑](#endnote-ref-33)
34. . Mathee A. Towards the prevention of lead exposure in South Africa: contemporary and emerging challenges. Neurotoxicology. 2014 Dec;45:220-3. doi: 10.1016/j.neuro.2014.07.007. Epub 2014 Jul 30. PMID: 25086205. [↑](#endnote-ref-34)
35. . WHO Regional Office for Africa. Lead exposure in African children. Contemporary Sources and concerns. 2015. [Available: who.int/iris/bitstream/handle/10665/200168/9780869707876.pdf;jsessionid=703889728E2730D68F9A93525A7B2650?sequence=1; accessed 4 March 2023]. [↑](#endnote-ref-35)
36. . USAID [U.S. Agency for International Development]. 2024. Press Release: Administrator Samantha Power Calls for a Global Effort To Eliminate Toxic Lead from Consumer Goods. usaid.gov/news-information/press-releases/jan-17-2024-administrator-samantha-power-calls-global-effort-eliminate-toxic-lead-consumer-goods; accessed 24 February 2024. [↑](#endnote-ref-36)
37. . G7 Workshop Summary Report. Lead as a Major Threat for Human Health and the Environment – An Integrated Approach Strengthening Cooperation Towards Solutions 9-10 November 2022, in Berlin, Germany and virtual. [Available: bmuv.de/fileadmin/Daten_BMU/Download_PDF/Chemikaliensicherheit/g7_workshop_report_lead_as_major_threat_bf.pdf; accessed 4 March 2023]. [↑](#endnote-ref-37)
38. . Lemière B. A review of pXRF (field portable X-ray fluorescence) applications for applied geochemistry. J Geochem Explor 2018;188:350-363. doi:10.1016%2Fj.gexplo.2018.02.006 [↑](#endnote-ref-38)
39. . FDA [U.S. Food and Drug Administration]. Update - February 15, 2023: Magellan Diagnostics resumed distribution of its LeadCare II test kits in February 2022, and resumed distribution of its LeadCare Plus and LeadCare Ultra test kits in October 2022. [Available: fda.gov/medical-devices/medical-device-recalls/magellan-diagnostics-recalls-leadcare-ii-leadcare-plus-and-leadcare-ultra-blood-lead-tests-due-risk; accessed 4 March 2023]. [↑](#endnote-ref-39)
40. . Dreibelbis R, Winch PJ, Leontsini E, Hulland KR, Ram PK, Unicomb L, Luby SP. The Integrated Behavioural Model for Water, Sanitation, and Hygiene: a systematic review of behavioural models and a framework for designing and evaluating behaviour change interventions in infrastructure-restricted settings. BMC Public Health. 2013 Oct 26;13:1015. doi: 10.1186/1471-2458-13-1015. PMID: 24160869; PMCID: PMC4231350. [↑](#endnote-ref-40)
